# Optimized Multiresidue Analysis of Organic Contaminants of Priority Concern in a Daily Consumed Fish (Grass Carp)

**DOI:** 10.1155/2017/9294024

**Published:** 2017-03-01

**Authors:** Wei He, Yanru Chen, Chen Yang, Wenxiu Liu, Xiangzhen Kong, Ning Qin, Qishuang He, Fuliu Xu

**Affiliations:** ^1^MOE Laboratory for Earth Surface Process, College of Urban & Environmental Sciences, Peking University, Beijing 100871, China; ^2^State Key Laboratory of Environmental Criteria and Risk Assessment, Chinese Research Academy of Environmental Sciences, Beijing 100012, China; ^3^Beijing Municipal Key Laboratory of Agriculture Environment Monitoring, Beijing 100097, China

## Abstract

The organic contaminants, including polycyclic aromatic hydrocarbons (PAHs), organochlorine pesticides (OCPs), polybrominated diphenyl ethers (PBDEs), and polychlorinated biphenyls (PCBs), are of priority concern because of their persistence, toxicity, and long-distance transportation in global environment. Their residues in a daily consumed fish (grass carp) pose potential threat to human health and aquatic ecosystems. The present study optimized an analytical protocol of microwave-assisted extraction (MAE), lip-removal by gel permeation chromatography (GPC), cleanup by solid phase cartridge (SC) or adsorption chromatography column (CC), and gas chromatography-mass spectrometry (GC/MS). Besides traditional statistical parameters, some indicators were calculated to judge the performances of extraction by various methods. The optimization experiment showed that n-hexane/acetone was the best MEA extraction solvent; an optimal fraction time of 10–39 min could simultaneously elute all the target chemicals in a single GPC run. Both CC and SC showed good recoveries. However, CC performed better than SC (*p* < 0.05) for OCPs, and SC performed better than CC for PBDEs (*p* < 0.05). We also emphasized the limitations and advantages of SC and CC and finally proposed SC as the promising cleanup method because of its low-cost materials, time-saving steps, being free of manual filling, and operation by automated SPE system.

## 1. Introduction

The organic contaminants of priority concern, including polycyclic aromatic hydrocarbons (PAHs), organochlorine pesticides (OCPs), polybrominated diphenyl ethers (PBDEs), and polychlorinated biphenyls (PCBs), are ubiquitous in the environment because of their persistence, toxicity, and long-distance atmospheric transport [1–3]. In aquatic ecosystems, OCPs, PBDEs, and PCBs tend to be biomagnified in the food chain. At the top level of the food chain, human beings who eat polluted fish are the terminal victims of those pollutants [4–6]. PAHs can damage DNA structures, cause chromosomal mutations, and increase risks of leukemia in children [7]. Some OCPs are endocrine disruption chemicals, which can cause reproductive diseases like breast cancer [8]. PBDEs can influence human thyroid secretion balance(s), neurological development, and endocrine and immune systems [9, 10]. PCBs can cause unusual lipid metabolism and depress the central nervous system [11, 12]. Although air and water are also major pathways of exposure to the above contaminants, their concentrations in these media are lower than in fish. Moreover, pollutants in air and water can be removed by current purification technologies before being inhaled and drunk, whereas it is very hard to remove those pollutants in fishes [13]. Those polluted fishes would be ingested by human beings and other animals. Therefore, from both ecological and human health points of view, it is important to determine and evaluate the risks of pollutants in fish [14]. The grass carp was chosen as our target fish due to its largest production (five million tons per year) and daily consumption throughout the world especially in China [15].

Typically, the determination procedure of pollutants in biological tissues commonly consists of extraction, cleanup, and instrument analyses. Extraction methods include Soxhlet extraction [26], ultrasonic extraction [27], pressurized liquid extraction (or accelerated solvent extraction) [17, 25, 28], supercritical fluid extraction [29, 30], and microwave-assisted extraction (MAE) [31]. Cleanup methods can be nondestructive or destructive [23]. The former consist of gel permeation chromatography (GPC) [19, 28, 32], adsorption chromatography (e.g., Florisil, silica, and alumina) [26], and solid phase extraction (SPE) (SPE cartridges) [23]. The sulfuric acid treatment is typically applied to the adsorption material, but this cleanup method is destructive [16]. In contrast, GPC followed by SPE cartridge cleanup is nondestructive and can be fully automated to completely and efficiently remove both lipids and interfering chemicals [33]. Gas chromatography (GC) combined with mass spectrometry (MS) is more widely used for separation, identification, and quantification of PAHs, OCPs, PBDEs, and PCBs than high-performance liquid chromatography (HPLC) coupled to a fluorescence detector or MS [2, 4, 18, 23, 25, 27–29, 32–37].

At present, most studies focus on determination of one group of PAHs, OCPs, PBDEs, and PCBs because their objectives were to figure out the sources and fate of a single group of chemicals [16, 19, 27, 31, 36]. However, to assess and compare the ingestion exposure and health risk of chemicals through eating fishes, we need to determine as more contaminants of priority concern as possible [38]. Thus, several researches attempt to perform multiresidue analysis of no more than three groups of above chemicals [23, 34]. The present study picked up the previous experience of multiresidue analysis, optimized it based on convincing and promising statistical tests and indicators, and proposed an MAE-GPC-SPE (or adsorption chromatography column) and GC/MS protocol for determination of PAHs, OCPs, PBDEs, and PCBs in a daily consumed fish (grass carp).

## 2. Materials and Methods

### 2.1. Fish Samples and Pretreatment

Because there are no reference materials of grass carp, which contains all the four groups of chemicals, the grass carp meat which was purchased from a local fish market in Beijing is used to mimic the organism matrix of grass carp. The fish was freeze-dried using a FDU-830 lyophilizer system (Tokyo Rikakikai Co., Ltd., Japan) to ensure adequate moisture removal after slaughtering, scaling, and cutting into small pieces and frozen at −20°C; the fish meat was lyophilized for approximately 2 days. Subsequently, the fish pieces were ground into powder using a MM400 mixer mill (Retsch Company, Germany), sealed with aluminum foil, and stored in a desiccator before analysis. Triplicate fish meat samples spiked with standard chemicals, fish meat blanks (without chemical spiking), and solvent blanks were analyzed in this study. The spiked meat samples were shaken for 12 hours to make the pollutants distribute into the meat tissue.

### 2.2. Reagents and Standards

Ethyl acetate (EA), cyclohexane (CH), n-hexane (HEX), acetone (ACE), and dichloromethane (DCM) were of HPLC grade and were supplied by J&K Chemical Ltd., in China. For GPC cleanup, Bio-Beads SX-3 (200–400 mesh) were purchased from Bio-Rad Laboratories GmbH (Munich, Germany). Anhydrous sodium sulfate (analytical grade), alumina (200–300 mesh and 300–400 mesh), silica gel (200–300 mesh), sodium hydroxide, and concentrated sulfuric acid were purchased from Sinopharm Chemical Reagent Co., Ltd., China. Silica gels (300–400 mesh) were purchased from Qingdao Haiyang Chemical Co., Ltd., China. All adsorption materials were baked at 450°C for 6 hr before use. Alumina and silica gels of 200–300 mesh size were used as adsorbents in the chromatographic column, whereas alumina and silica gels of 300–400 mesh size were used as adsorbents in the SPE cartridge. Both the alumina and silica gel were reactivated for 16 hr at 130°C before use. 3 g of ultrapure water was added to 100 g of cooled silica gel or alumina to change its polarity and they were called neutral silica gel(s) and alumina. 33 g of 1.0 mol L^−1^ sodium hydroxide and 44 g of concentrated sulfuric acid were added to 100 g of silica gel to form alkaline silica gel and acidic silica gel, respectively. For the wet method of packing the chromatography column, HEX was added to the alumina and silica gels, which were then soaked overnight. All glassware, including columns, flasks, bottles, vial insert tubes, and vials, were ultrasonically cleaned with detergent, washed with ultrapure water, baked at 450°C for 6 hr, and rinsed with ACE twice. The microwave extract ion cylinders, plugs, and lids were ultrasonically cleaned with distilled water, washed with ultrapure water, rinsed with ACE twice, and placed on a filter paper to dry.

The twenty-eight PAHs included the original 16 PAHs under priority control by US Environmental Protection Agency (EPA), 8 PAHs like benzo[c]phenanthrene, cyclopenta[c,d]pyrene, benzo[e]pyrene, dibenzo[a,e]pyrene, dibenzo[a,i]pyrene, dibenzo[a,h]pyrene, dibenzo[a,l]pyrene, and dibenzo[a,e]fluoranthene frequently monitored according to recommendations by the EU Scientific Committee for Food, the European Union, Quebec Ministry of Environment, and the US EPA, and 4 PAHs like perylene, anthanthrene, retene, and coronene frequently found in ambient air samples based on USEPA's research and literature surveys [39]. The PAH surrogate standards (SSs) were 2-Fluorobiphenyl and p-Terphenyl-d14, and its internal standards (ISs) were acenaphthene-d10, anthracene-d10, chrysene-d12, naphthalene-d8, and perylene-d12 (AccuStandard, Inc., NY, US). The twenty-five OCPs (AccuStandard, Inc., NY, US) used in this study were as follows: aldrin;* cis*-chlordane;* trans*-chlordane; oxychlordane; o,p′-DDD; p,p′-DDD; o,p′-DDE; p,p′-DDE; o,p′-DDT; p,p′-DDT; dieldrin; endosulfan I; endosulfan II (ES II); endrin; *α*-hexachlorocyclohexane (*α*-HCH); *β*-HCH; *γ*-HCH; *δ*-HCH; heptachlor;* cis*-heptachlor epoxide (cHCP);* trans*-heptachlor epoxide (tHCP); hexachlorobenzene (HCB); isodrin; methoxychlor (MOC); and mirex. The SS was 1-bromo-2-nitrobenzene (OCP-SS), and the IS was pentachloronitrobenzene (OCP-IS).

The fourteen PBDEs (AccuStandard, Inc., NY, US) consisted of the following congener numbers: PBDE-17, PBDE-28, PBDE-47, PBDE-66, PBDE-71, PBDE-85, PBDE-99, PBDE-100, PBDE-138, PBDE-153, PBDE-154, PBDE-183, PBDE-190, and PBDE-209. The PCBs (AccuStandard, Inc., NY, US) include PCB-18, PCB-37, PCB-44, PCB-49, PCB-52, PCB-70, PCB-74, PCB-77, PCB-81, PCB-87, PCB-99, PCB-101, PCB-105, PCB-114, PCB-118, PCB-119, PCB-123, PCB-126, PCB-128, PCB-138, PCB-151, PCB-153, PCB-156, PCB-157, PCB-158, PCB-167, PCB-168, PCB-169, PCB-170, PCB-177, PCB-180, PCB-183, PCB-187, PCB-189, PCB-194, and PCB-199. Considering the cost factor and similar property between PBDEs and PCBs, the PBDEs and PCBs had the same SS and IS, which were 2,2′,3,4,5,5′-hexaCB (^13^C_12_, 99%, EC-1418-1.2, Cambridge Isotope Laboratories, Inc., USA) (141) and 2,2′,3,3′,4,5,5′,6,6′-nonaCB (^13^C_12_, 99%, EC-1419-1.2, Cambridge Isotope Laboratories, Inc., USA) (208), respectively. More information about the standards is shown in Tables S1–S4 in Supplementary Material available online at https://doi.org/10.1155/2017/9294024.

### 2.3. MAE

Approximately 2.0 g of fish powder was weighed and loaded into the extraction cylinders. A certain amount of SSs of each chemical type was added to the cylinders (200 ng for PAHs, 100 ng for OCPs, and 50 ng for PCBs and PBDEs), after which 25 mL extract solvent was loaded. The MAE was performed by a MARS 5 Microware Accelerated Reaction System (CEM Corp., Matthews, NC, USA) with the following instrument conditions: working power was 1,200 W; temperature increased to 100°C within 10 min; then temperature held at 100°C for 10 min; and then temperature decreased to ambient temperature within 30 min. After extraction, the matrices and solvent were filtered through glass fiber filters (baked at 450°C for 6 hr before use). The filtered extract was concentrated to approximately 1 mL using a N-1100D-WD rotary evaporator (Tokyo Physicochemical Corporation, Japan), after which 10 mL EA/CH (1 : 1, v/v) was added. Subsequently, the solvent was concentrated to approximately 2 mL and then centrifuged using high-speed refrigerated centrifugation (12000 r min^−1^) for 15 min at 4°C (5417R, Eppendorf Corporation, Germany). The supernatant was filtered through a 0.45 *μ*m Teflon microporous filter before GPC cleanup. To choose the best extract solvent in MAE, three solvent mixtures, EA/CH (1 : 1, v/v, ES1), DCM/HEX (4 : 1, v/v, ES2), and HEX/ACE (1 : 1, v/v, ES3), were chosen to extract blank and spiked fish meat.

### 2.4. GPC Cleanup

The GPC mobile phase included DCM/HEX, DCM, and EA/CH in previous studies [16, 19, 25, 28, 32–34]. In the present study, we employed the EA/CH (1 : 1, v/v) because it had been proven to elute PAHs, PCBs, and OCPs from Bio-Beads SX-3 column in a single run [17, 34]. The EA/CH was ultrasonically treated for 30 min to remove air bubbles before use. An auto-GPC system, GPC800+ (LabTech Holdings, Inc., Beijing, China) with column (Bio-Beads SX-3, 300 × 20 mm), was used for GPC cleanup employing a flow rate of 5 mL min^−1^ and a full loop injection of 2 mL. To determine the GPC fraction times of various target chemicals, fish meat microwave extracts spiked with 2 mg L^−1^ of PAHs, OCPs, PBDEs, and PCBs (including IS and SS) were automatically injected. Macromolecules, such as lipids and proteins, were thoroughly separated within 0–10 min, after which 10 fractions were obtained (15 mL per fraction for PAHs and 10 mL per fraction for the other three constituents). The fractions were concentrated to 1 mL, exchanged with 10 mL HEX, and further concentrated to 1 mL before instrument analysis.

### 2.5. SPE Cartridge Cleanup

An SPE cartridge containing only neutral silica gel or both neutral alumina and neutral silica showed good cleanup effect for PAHs, OCPs, PBDEs, and PCBs [34]. However, the trace PBDEs and PCBs might be disturbed by some impurities (e.g., fatty acids), which were simultaneously eluted with PBDEs, when analyzed using negative chemical ionization (NCI) source of GC-MS. Hence, acid silica gel is typically filled in the SPE cartridge to remove those impurities [16, 33]. In the present study, as an alternative strategy, the SPE cartridge containing 250 mg of neutral alumina, 500 mg of acid silica gel, and 250 mg of neutral silica gel (from bottom to top) was used for analysis of PBDEs and PCBs. To soak the adsorbent, 20 mL of HEX was added to the cartridge, after which the GPC eluate was loaded onto the cartridge. To determine the optimum elution solvent and its volume, DCM, DCM/HEX (1 : 1, v/v) (MIX), and HEX were used to elute the blank cartridges spiked directly with SSs and analytes with SSs on the top of three independent SPE silica gel cartridges (one for each elution solvent). For each solvent, 10 fractions (2 mL per fraction) were collected, followed by solvent exchange with 10 mL HEX, concentration to 1 mL, and addition of ISs before instrument analysis.

### 2.6. Chromatography Column Cleanup

When the target compounds were PAHs and OCPs, the GPC eluate was loaded onto a multilayer column (inner diameter = 10 mm) that was packed from bottom to top with 12 cm wet neutral alumina, 12 cm wet neutral silica gel, and 1 cm anhydrous sodium sulfate. After adding (and discarding) 10 mL HEX, the eluate was eluted with 50 mL MIX. When the target compounds were PBDEs and PCBs, the GPC eluate was loaded onto a multilayer column (inner diameter = 10 mm) packed from bottom to top with 6 cm wet neutral alumina, 2 cm wet neutral silica gel, 5 cm wet alkaline silica gel, 2 cm wet neutral silica gel, 6 cm wet acidic silica gel, and 1 cm anhydrous sodium sulfate. The eluate was eluted with 70 mL MIX. The subsequent steps were the same as those used in the SPE cartridge cleanup.

### 2.7. Instrument Analysis

The eluate from cartridge or column was concentrated to 1 mL. After a solvent exchange using 10 mL HEX, the eluate was concentrated to 1 mL, was added into with ISs, evaporated using nitrogen gas, finally concentrated to 100 *μ*L, and stored in vials at −20°C before analysis. The GC/MS or GC/MS/MS used in our laboratory consists of four instruments that are specific for analysis of PAHs, OCPs, PBDEs, and PCBs, respectively. The instrument conditions are listed in Text S1, and the ions selected for identification/quantification as well as the instrument limit(s) of detection are summarized in Tables S1–S4.

### 2.8. Data Handling

To obtain the best MAE solvents, we employed one-way analysis of variance (ANOVA) and the independent sample test (IST). However, test of homogeneity of variances (*p* > 0.05) should be done to tell whether ANOVA could be performed for the dataset. If ANOVA could not be performed as indicated by *p* < 0.05, IST was alternatively used to distinguish the differences between two datasets. Levene's test typically determined the homogeneity (*p* > 0.05) and inhomogeneity (*p* < 0.05) of dataset's variance. IST offers two results corresponding to homogeneous and inhomogeneous datasets, respectively.

Several terms, *R*_ANOVA_, ANOVA^*U*^, ANOVA^*S*^, *R*_IST_, IST^*U*^, and IST^*S*^, were introduced to overall compare the three MAE solvents. Assuming that one chemical group like PCBs had *N* congeners and the ANOVA could be performed for *N*_ANOVA_ congeners as indicated by *p* > 0.05 calculated by test of homogeneity of variances, ratio of congeners with “qualified” (*p* > 0.05) ANOVA to total congeners in specific chemical group can be calculated by the following equation:

(1) $$\begin{array}{c} R_{ANOVA}=\frac{N_{ANOVA}}{N}. \end{array}$$

After homogeneity of variances test, the ANOVA was performed for the “qualified” *N*_ANOVA_ congeners and no significance among the three solvent mixtures was found for *N*_ANOVA_^*U*^ congeners. ANOVA^*U*^ was calculated by (2), indicating the ratio of *N*_ANOVA_^*U*^ to *N*_ANOVA_. And significance among the solvent mixtures was found for *N*_ANOVA_^*S*^ congeners. Multiple comparative analysis was performed to find the best solvent mixture. And those congeners were listed in the “ANOVA^*S*^” column of Table 1.

(2) $$\begin{array}{c} {ANOVA}^{U}=\frac{{N_{ANOVA}}^{U}}{N_{ANOVA}}. \end{array}$$

Similar to *R*_ANOVA_, *R*_IST_ was calculated by dividing *N*_IST_ congeners, which obeyed normal distribution, by the total number of congeners *N* in the following equation:

(3) $$\begin{array}{c} R_{IST}=\frac{N_{IST}}{N}. \end{array}$$

After Levene's test was performed, IST multiple comparison was conducted among the three solvent mixtures. Whether the dataset is homogeneous or not, if three *p* values of the three combinations calculated by IST were >0.05, that indicated that there was no significance difference among them. This result was found for *N*_IST_^*U*^ congeners, and IST^*U*^ was calculated by (4). The congeners, which had significantly different extract ion effect by three solvent mixtures, were listed in the “IST^*S*^” column in Table 1. And the rank by extract ion effect was also shown in that column.

(4) $$\begin{array}{c} {IST}^{U}=\frac{{N_{IST}}^{U}}{N_{IST}}. \end{array}$$

## 3. Results and Discussion

### 3.1. Extract Solvent Selection for MAE

Of the three alternative extraction solvents, DCM/HEX (4 : 1, v/v) was slightly modified following previous study [40]. HEX/ACE (1 : 1, v/v) is recommended by US EPA Method 3546 and proposed by many studies [22, 41]. The GPC mobile phase (EA/CH) was also an alternative because the number of solvent types could be reduced if it was suitable for the extraction of all target chemicals. Three extraction mixture solvents have slightly different composite octanol-water partition coefficient (log_10_*K*_OW_): EA/CH (1 : 1), 1.03, DCM/HEX (4 : 1), 1.30, and HEX/ACE (1 : 1), 0.59 [42]. However, there were no significant extraction differences among the three solvent mixtures for more than 60% of target chemicals in the blank fish as indicated by IST^*U*^ column in Table 1 (also see originally statistical analysis in Tables S5). Nevertheless, we still could observe some slight extraction differences: (1) HEX/ACE appeared to extract more PCBs (PCB-52, PCB-87, and PCB-99) and OCPs (HCB) as indicated by the cases' comparison in IST^*S*^ column in Table 1, whereas (2) DCM/HEX was demonstrated to be the best extraction mixture for PBDE-209. Based on the similarity-intermiscibility theory, PCB-52, PCB-87, and PCB-99 have relatively lower *K*_OW_ among the PCB congeners. And HEX/ACE also has relative lower *K*_OW_ than other solvents. The DCM/HEX's performance for PBDE-209 could also be explained by the interaction between solvent and target chemicals.

In the spiked fish meat, the three solvents' extraction effect showed no significant differences for more than 80% of target chemicals (Table 1 and Table S6). The solvents selectively extract some target chemicals: (1) DCM/HEX could extract the most benzo[a]pyrene (*p* < 0.05); (2) HEX/ACE could extract the most OCP-SS (*p* < 0.05); and (3) both DCM/HEX and HEX/ACE had similarly better extraction performance for BDE-SS and 7 PCB congeners than EA/CH. In practice, the recoveries of target chemicals by various solvents are also of great concern when optimizing an analytical method [9, 25, 33]. Like the extraction concentration, there were no significant differences in recoveries when using the three solvents for more than 80% of the four groups of chemicals (*p* > 0.05) (Table 1 and Table S7). Still, we found that EA/CH had the best recoveries for BDE-SS, PCB-183, and PCB-189 (*p* < 0.05) and the worst recoveries for 4 PCB congeners (*p* < 0.05); HEX/ACE had the best recoveries for OCP-SS and o,p′-DDT (*p* < 0.05) and the worst recoveries for PCB-183 and PCB-189 (*p* < 0.05); and DCM/HEX had the worst recoveries for acenaphthene, OCP-SS, ES II, and o,p′-DDT (*p* < 0.05). Quality judgment (QJ) criteria in US EPA methods are often set at 70–130% recovery values with <30% relative standard deviation (RSD) values [43]. We employed this criterion to determine the best solvent for a particular chemical group, as shown in Table S8. For 93.75% of PAHs and 100% of PBDEs, HEX/ACE was the proposed solvent, whereas for OCPs and PCBs there were no significant differences in recoveries among the three solvents. In conclusion, all three solvents were suitable for extraction of nearly all the target chemicals. However, HEX/ACE was recommended as the MAE solvent in our analytical procedure due to its best performance based on comprehensive analysis above.

### 3.2. GPC Fraction Time

The GC/MS determinations of target chemicals at every GPC fraction from the 10th min are compiled in Table S9. The fraction times are as follows: 10 to 34 min for PAHs, 10 to 20 min for OCPs, 10 to 24 min for PBDEs, and 10 to 18 min for PCBs. Organic halogen compounds had shorter fraction times than PAHs. To simultaneously elute all the target chemicals in a single GPC run, a longer fraction time of 10 to 34 min was chosen. Five additional minutes were added to ensure sufficient collection of the target chemicals. Thus, our experimental procedure employed a fraction time of 10 to 39 min.

### 3.3. SPE Elution Solvent

**Figure 1:**
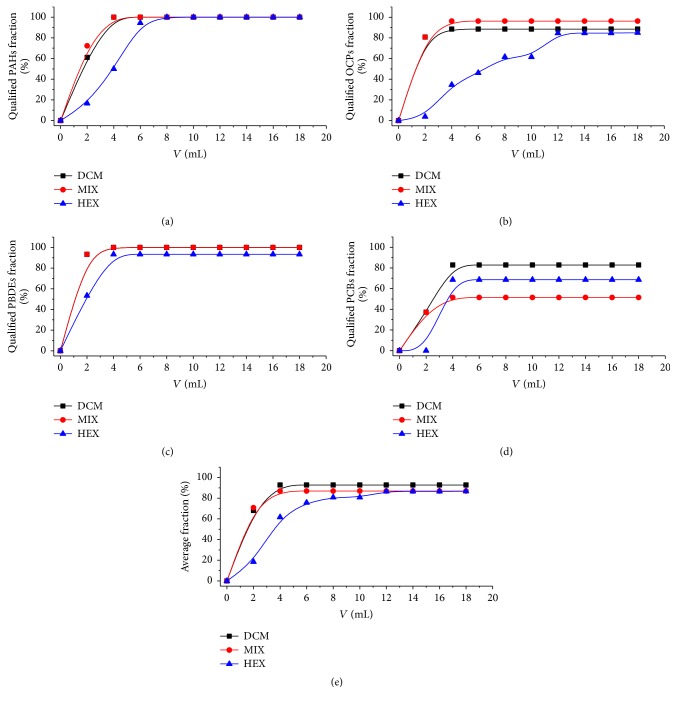
The fraction of qualified target chemicals (cumulative recovery value > 70%) with increase of elute volume. The charts (a, b, c, d, and e) denote PAHs, OCPs, PBDEs, PCBs, and average fraction of those four groups of target chemicals.

The silica gel or alumina typically retains the polar impurity in concentrated eluent from GPC [28]. The nonpolar target chemicals are generally eluted by nonpolar solvents such as HEX and DCM [19, 23, 25, 34]. HEX is preferentially used to extract or elute the nonpolar organics with straight chain such as n-alkane or n-olefin [44]. On the contrary, DCM prefers to affine with organics with branched structure such as aromatics, ethers, and organic halogens (e.g., R-Cl, R-Br) [17, 25, 26]. In the present study, the cumulative recoveries with increase of solvents for each specific chemical were summarized in Figure 1 with details in Figures S1–S4. For all target chemicals, the cumulative recoveries obtained at 6 mL using DCM or MIX approached 70%–100%. However, when using HEX, cumulative recoveries obtained at 8 mL and 10 mL approached 100% for PAHs with 2-3 aromatic rings and 4-5 aromatic rings, respectively. Regarding OCPs, it was difficult to elute many chemicals (such as *δ*-HCH, cHCP, tHCP, and ES II) from the SPE cartridge with HEX. The three solvents exhibited similar elution effects for PBDEs; however, it was difficult to elute PBDE-190 using HEX. Regarding PCBs, many congeners (such as PCB-74, PCB-77, and PCB-126) were difficult to be eluted with any of the solvents. According to Figure 1, DCM was the strongest elution solvent, as demonstrated by the fact that only 8 mL DCM could elute most target chemicals. The next strongest elution solvent was MIX. HEX was so weak that over 20 mL HEX could not thoroughly elute OCPs. Given that the number of matrix chemicals eluted by DCM and MIX approached that of those eluted by DCM, we chose MIX as the most efficient solvent and employed a volume of 15 mL. Moreover, the solvent system of sample should be changed to HEX before GC/MS analysis. Thus, we tried to use less volume of DCM in the elution.

### 3.4. Cleanup Effect of SPE Cartridges by Comparison with Traditional Chromatography Column

**Figure 2:**
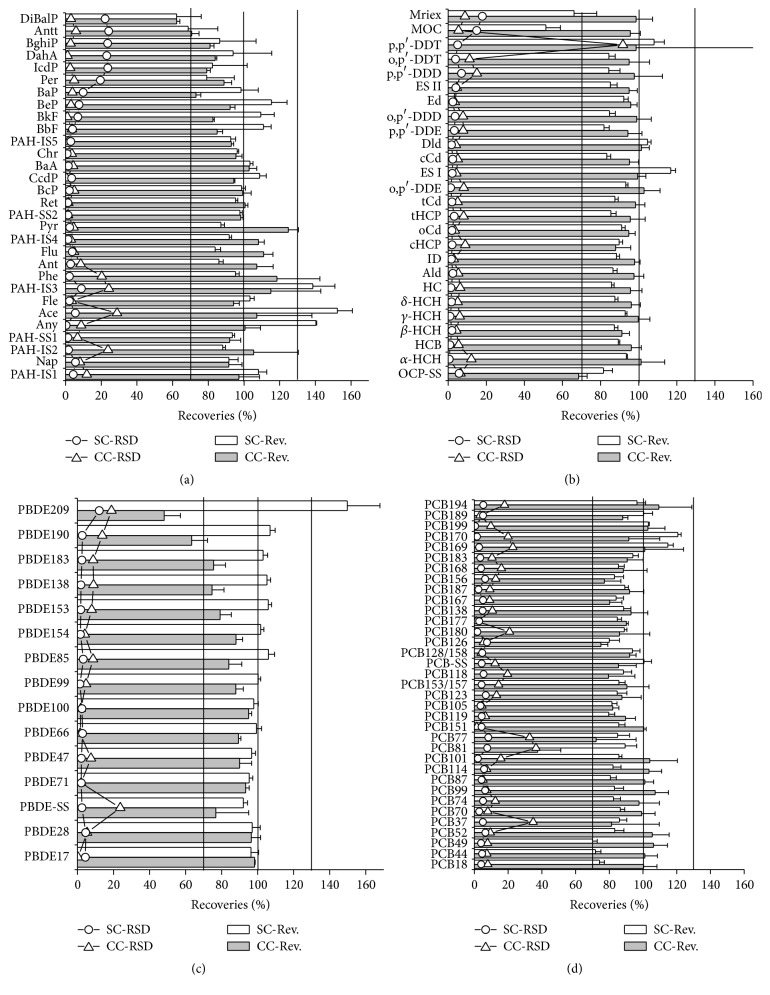
Recoveries (RV, the column), their standard deviation (SD, the error bar), and their relative standard deviation (RSD, the line and empty symbol) of PAHs (chart a), OCPs (chart b), PBDEs (chart c), and PCBs (chart d) in spiked fish meat cleaned up by SPE cartridge (SC, the white column and the empty circle) and chromatography column (CC, the grey column and the empty triangle), respectively.

The recoveries, standard deviation values, and relative standard deviation values of PAHs, OCPs, PBDEs, and PCBs in spiked fish meat cleaned up via SPE cartridge (SC) and chromatography column (CC), respectively, are compared in Figure 2. For PAHs, regardless of whether cleanup was performed via SC or CC, PAHs with more rings tended to have lower recoveries. Neither of the two cleanup methods was superior for DiBalP. For OCPs, nearly all target chemicals' recoveries were in the acceptable range of 70%–100%, except for mirex and MOC when cleaned up by SC and OCP-SS when cleaned up by CC. For PBDEs, SC provided similar recoveries for each congener, except for PBDE-209, whereas congeners containing more bromine had lower recoveries when cleaned up by CC. For PCBs, almost all congeners had acceptable recoveries except for PCB-81 when cleaned up by CC.

To further compare the recoveries of target chemicals obtained with SC versus CC, ANOVA and IST tests were performed in Table S10 and summarized in Table 2. There were no significant differences between recoveries obtained via cleanup by SC versus CC for 60% of PAHs, 57.7% of OCPs, 33.3% of PBDEs, and 68.6% of PCBs (IST, *p* > 0.05). It may appear that the recoveries of PAHs cleaned up by SC (98.6 ± 19.4%) were higher than those cleaned up by CC (95.1 ± 14.3%); however, considering the number of target chemicals with the better recovery for a specific cleanup method as indicated by IST, SC and CC were both good for 18 of 28 PAHs, and SC and CC offered better recovery for 5 of 28 PAHs and 7 of 28 PAHs, respectively, indicating that CC might be better. Specifically, CC provided much better recovery for PAHs with 5-6 aromatic rings like indeno[1,2,3-cd]pyrene, benzo[ghi]perylene, and anthanthrene, whereas SC provides much better recovery for PAHs with 2–4 aromatic rings like fluorene, retene, benzo[c]phenanthrene, chrysene, and benzo[k]fluoranthene. Cleanup by CC provided better recoveries for OCPs (95.7 ± 6.4%) (*p* < 0.05). Likewise, CC cleanup did not provide a significantly higher recovery for any of the PBDE congeners (*p* < 0.05). For PCBs, only two congeners (PCB-81 and PCB-189) had better recoveries when cleaned up solely by SC. In conclusion, the two cleanup methods considered herein provided similar recoveries for most of the target chemicals considered. For PAHs, both CC and SC provided good recoveries; on the other hand, CC performed better for OCPs and PCBs, and SC performed better for PBDEs.

### 3.5. Proposed Analytical Method Scheme

**Figure 3:**
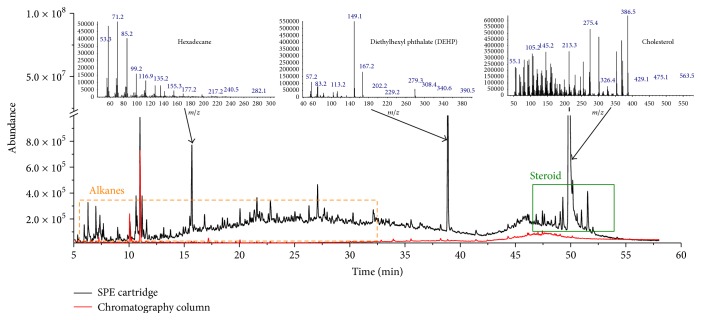
GC/MS scan of spiked fish meat cleaned up using an SPE cartridge and a chromatography column (the box with dash border denotes that impurities in this area are mainly alkanes, and the box with solid border denotes that impurities in this area are mainly steroids).

As shown in Figure 3, GC/MS scans of spiked fish meat cleaned up by SC and CC indicated that SC brought more interferences to the signal than CC. Alkanes (n-hexadecane), phthalate esters (diethylhexyl phthalate), and steroids (cholesterol) were all detected in fish samples cleaned by SC. Although the adsorbent with mesh size of 300–400 used in SC was stronger than that used in CC with mesh size of 200–300, the total adsorption capacity of SC was less than that of CC in our study when considering their different usage amounts. Upon reaching saturation, the SC capacity was not efficient in the removal of some small molecules. The CC cleanup method was also proposed because it prolonged the life of the GC/MS capillary chromatography column, microsyringe, and ion source. On the other hand, SC was also desirable because it could reduce elution time and save solvent [28]. Because commercial products of SPE are often provided, we could also avoid filling the cartridge. More importantly, using the automated SPE system after the automated GPC system reduced time and effort [33]. Although it seemed that both SC and CC had their own advantages and limitations, we finally proposed CC as the promising cleanup method because of its low-cost materials, time-saving steps, being free of manual filling, and operation by automated SPE system.

**Figure 4:**
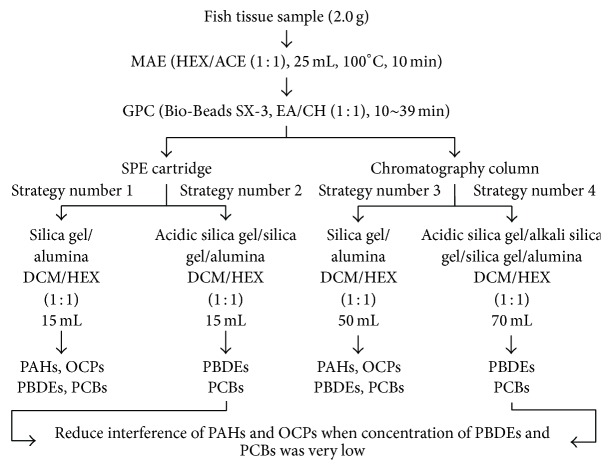
Proposed analytical method scheme.

**Figure 5:**
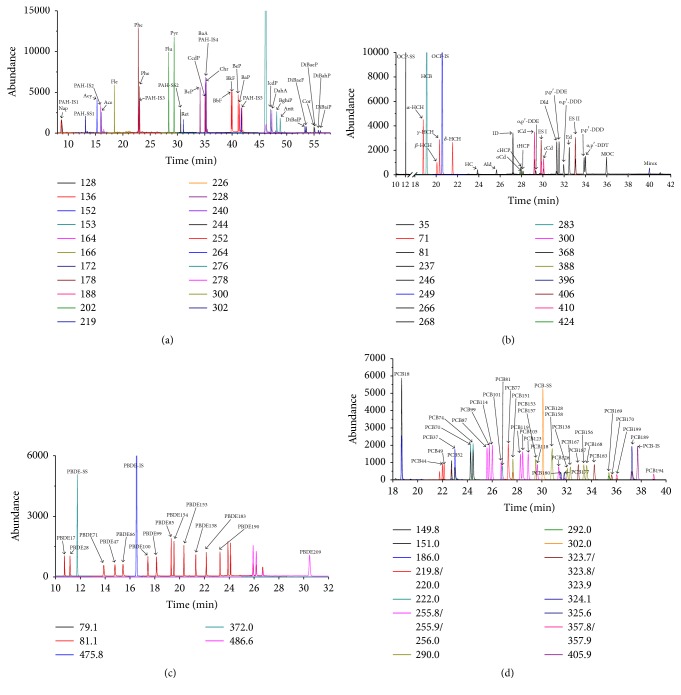
GC/MS or GC/MS/MS chromatogram for the analysis of fish meat tissue spiked with PAHs (chart a) at 100 ng g^−1^, OCPs (chart b) at 50 ng g^−1^, PBDEs (chart c) at 25 ng g^−1^ (PBDE 209 at 125 ng g^−1^), and PCBs (chart d) at 25 ng g^−1^ using scheme #1 in Figure 4.

The proposed analytical methodology, summarized in Figure 4, was determined based on the results and considerations discussed above. 2.0 g of fish samples was extracted with 25 mL HEX/ACE (1 : 1) by MAE, followed by GPC cleanup with time range from 10 to 39 min. Four strategies were employed according to cleanup effect: the 1st and 3rd strategies could provide simultaneous cleanup and determination of PAHs, OCPs, PBDEs, and PCBs; the former might save time and solvents but cause interferences and shorten the life of the GC/MS consumables, and the latter would prolong the life of the GC/MS consumables but cost a lot; the 2nd and 4th strategies could remove some larger molecules, which were not thoroughly cleaned up by GPC, and small molecules like PAHs and OCPs [16]; sometimes the fish might be polluted by impurities (e.g., fatty acids and pigments) with high concentration; these strategies would reduce interference of those impurities and offered credible determination of trace PBDEs and PCBs. Using scheme #1 in Figure 4, fish samples spiked with PAHs (100 ng g^−1^), OCPs (50 ng g^−1^), PBDEs (25 ng g^−1^), and PCBs (25 ng g^−1^) (PBDE-209 at 125 ng g^−1^) were analyzed by GC/MS or GC/MS/MS (chromatogram shown in Figure 5).

Compared with previous studies in Table 3, the proposed analytical protocol incorporates some advanced methods, including MAE extraction, GPC cleanup, and SPE cleanup. That analytical protocol allowed us to determine four groups of chemicals, whereas many previous studies only involved no more than three groups [23, 25]. Our lab has developed GC-MS instrument methods for those four groups of chemicals separately. PAHs and PCBs were quantified using GC/MS electron impact with selective ion monitoring mode (EI-SIM) and PBDEs and OCPs were quantified using negative chemicals ionization with selective ion monitoring mode (NCI-SIM) as recommended by previous studies (Table 3 and Text S1) [16, 18, 25]. Generally, lower detection limits of OCPs could be obtained by NCI-SIM rather than EI-SIM and it is hard to detect and quantify PAHs using NCI-SIM [18, 19, 25]. If we selected EI-SIM, the resolution and sensitivity of our GC-MS instruments were not so high to quantify all those four groups of chemicals with low detection limits. Still, we realized that it was time-consuming and costly to analyze those target chemicals by different instruments. The present work could be further improved by developing instrument analysis which allows multiple residue analyses. Because ultrahigh resolution GC/TOFMS EI-SIM had offered good separation, identification, and quantification for PAHs, PBDEs, and PCBs, our next step was to employ ultrahigh resolution GC/TOFMS EI-SIM with multiple column chromatography to separate, identify, and quantify PAHs, OCPs, PBDEs, and PCBs in a single run [23, 45]. Considering the fish mass (2.0 g), concentrated eluent (100 *μ*L) before determination, and instrument limit of detection (LOD, 3 times of signal-to-noise ratio) in Tables S1–S4, limit of quantitation (LOQ, 10 times of signal-to-noise ratio) of PAHs, OCPs, PBDEs, and PCBs was calculated as follows: 0.08–0.33 ng g^−1^, 0.01–0.83 ng g^−1^, 0.08–0.42 ng g^−1^, and 0.08–0.83 ng g^−1^, respectively, which were similar to another research [23]. This method could be potentially used in other carps, which had similar lipid content to grass carp [20].

## 4. Conclusions

Based on the statistical analysis, our multiresidue analysis of organic contaminants of priority concern included the following: (1) the HEX/ACE was selected as the MEA extraction solvent due to its best performance among EA/CH, DCM/HEX (4 : 1, v/v), and HEX/ACE; (2) a fraction time of 10–39 min was taken to simultaneously elute all the target chemicals in a single GPC run using EA/CH solvent; (3) SC with an elution solvent of 15 mL MIX (1 : 1, v/v) was selected to clean the impurity up. The recoveries and full MS scan of eluent cleaned up by CC and SC showed that the two cleanup methods offered similar recoveries for most target chemicals. For PAHs and PCBs, both CC and SC showed good recoveries. For OCPs, CC performed better than SC (*p* < 0.05). On the other hand, SC performed better than CC for PBDEs (*p* < 0.05). Although both SC and CC had unique advantages, SC was finally proposed due to its low-cost materials, time-saving steps, being free of manual filling, and operation by automated SPE system.

## Supplementary Material

The supplemental materials with 30 pages include the instrument conditions of GC/MS matrix in Text S1, fragment ions for identification/quantification plus instrument limit of detection (LOD) for target chemicals in Table S1–S4, ANOVA and IST analysis of extracted target chemicals in blank and spiked fish by EA/CH (1:1), DCM/HEX (4:1), and HEX/ACE (1:1) in Table S5–S7, recoveries (RV), relative standard deviation (RSD), quality judgment (QJ) of target chemicals in spiked fish meat extracted by EA/CH (1:1), DCM/HEX (4:1), and HEX/ACE (1:1) in Table S8, GC/MS abundance of target chemicals at every fraction of GPC from the 10th min in Table S9, ANOVA and IST analysis of recoveries of target chemicals in spiked fish meat cleaned up by SPE cartridge (SC) and chromatography column (CC) in Table S10, recoveries of target chemicals at each SPE eluate when using DCM, DCM/HEX (1:1), HEX in Figures S1–S4, and recoveries with their standard deviation and their relative standard deviation of target chemicals in spiked fish meat cleaned up by SPE cartridge and chromatography column.

## Figures and Tables

**Table 1:** ANOVA and IST analysis of extracted PAHs, OCPs, PBDEs, and PCBs by EA/CH (1 : 1) (ES1), DCM/HEX (4 : 1) (ES2), and HEX/ACE (1 : 1) (ES3) in blank fish meat and spiked fish meat.

Groups	*R*_ANOVA_, %	ANOVA^*U*^, %	ANOVA^*S*^	*R*_IST_, %	IST^*U*^, %	IST^S^
Blank fish meat without SS (concentration of the target chemicals, “>” means better, “≈” means similar)						
PAHs	56.3	100.0		93.8	100.0	
OCPs	44.0	90.9	HCB, ES1 ≈ ES3 > ES2	52.0	100.0	
PBDEs	35.7	100.0		35.7	80.0	BDE209, ES2 > ES1 ≈ ES3
PCBs	20.6	100.0		41.2	64.3	PCB52, ES3 > ES1 ≈ ES2
						PCB87, ES3 > ES1 ≈ ES2
						PCB99, ES3 > ES1 ≈ ES2

Spiked fish meat with SS (concentration of the target chemicals, “>” means better, “≈” means similar)						
PAHs	66.7	100.0		100.0	94.4	BaP, ES2 > ES1 ≈ ES3
OCPs	80.8	95.2	OCP-SS, ES3 > ES1 ≈ ES2	100.0	88.5	OCP-SS, ES3 > ES1 ≈ ES2
						ES II, ES1 ≈ ES3 > ES2
						o,p′-DDT, ES1 ≈ ES2 > ES3
PBDEs	100.0	100.0		100.0	93.3	BDE-SS, ES2 ≈ ES3 > ES1
PCBs	100.0	94.3	PCB187, ES2 ≈ ES3 > ES1	100.0	80.0	PCB74, ES2 ≈ ES3 > ES1
			PCB189, ES2 ≈ ES3 > ES1			PCB99, ES2 ≈ ES3 > ES1
						PCB138, ES2 ≈ ES3 > ES1
						PCB187, ES2 ≈ ES3 > ES1
						PCB168, ES2 ≈ ES3 > ES1
						PCB183, ES2 ≈ ES3 > ES1
						PCB189, ES2 ≈ ES3 > ES1

Spiked fish meat with SS (recoveries of the target chemicals, “>” means better, “≈” means similar)						
PAHs	66.7	100.0		100.0	94.4	Ace, ES3 ≈ ES1 > ES2
OCPs	80.8	95.2	OCP-SS, ES3 > ES1 ≈ ES2	100.0	88.5	OCP-SS, ES3 > ES1 ≈ ES2
						ES II, ES1 ≈ ES3 > ES2
						o,p′-DDT, ES3 > ES1 ≈ ES2
PBDEs	100.0	100.0		100.0	93.3	BDE-SS, ES1 > ES3 ≈ ES2
PCBs	100.0	94.3	PCB187, ES2 ≈ ES3 > ES1	100.0	82.9	PCB74, ES3 ≈ ES2 > ES1
			PCB189, ES1 > ES2 ≈ ES3			PCB138, ES2 ≈ ES3 > ES1
						PCB187, ES3 ≈ ES2 > ES1
						PCB168, ES2 ≈ ES3 > ES1
						PCB183, ES1 > ES2 ≈ ES3
						PCB189, ES1 > ES2 ≈ ES3

Notes: *R*_ANOVA_, ANOVA^*U*^, ANOVA^*S*^, *R*_IST_, IST^*U*^, and IST^*S*^ were described in Section 2.8 in detail. The target chemicals, whose three solvents' extractions were significantly different, were listed in ANOVA^*S*^ and IST^*S*^ columns based on ANOVA's and IST's multiple comparative tests.

**Table 2:** Comparison of recoveries and relative standard deviation of PAHs, OCPs, PBDEs, and PCBs by SPE cartridge (SC) cleanup and chromatography column (CC) cleanup.

	PAHs		OCPs		PBDEs		PCBs	
SPE cartridge cleanup								
	RV, %	RSD, %	RV, %	RSD, %	RV, %	RSD, %	RV, %	RSD, %
AM	98.6	7.4	87.8	3.7	103.6	3.2	87.8	4.5
SD	19.4	8.2	12.0	4.1	13.5	2.6	10.4	1.8
CV	0.196	1.114	0.137	1.086	0.131	0.811	0.119	0.392
MD	95.3	3.6	87.4	2.3	100.3	2.5	85.6	4.4

Chromatography column cleanup								
	RV, %	RSD, %	RV, %	RSD, %	RV, %	RSD, %	RV, %	RSD, %
AM	95.1	6.9	95.7	9.9	82.7	7.9	91.1	12.5
SD	14.3	7.5	6.4	16.9	13.6	6.6	13.6	8.8
CV	0.150	1.094	0.067	1.708	0.164	0.843	0.149	0.701
MD	94.9	4.0	96.1	5.8	87.9	7.5	91.6	9.8

ANOVA test between SC and CC								
*p*	0.394		0.004		0.001		0.244	

The number of the cleanup methods with the better recovery as indicated by IST								
	Number (ratio, %)		Number (ratio, %)		Number (ratio, %)		Number (ratio, %)	
*N*_*U*_	18 (60.0)		15 (57.7)		5 (33.3)		24 (68.6)	
*N*_SC_	5 (16.7)		0 (0.0)		9 (60.0)		2 (5.7)	
*N*_CC_	7 (23.3)		11 (42.3)		1 (6.7)		9 (25.7)	

Notes: AM, SD, CV, and MD denoted the arithmetic mean, the standard deviation, the coefficient of variation, and the median. In “QJ” column in Table S10, “*U*” denoted that there were no significant differences between recoveries of the two cleanup methods; “CC” denoted that chromatography column cleanup method was better; “SC” denoted that SPE cartridge cleanup method was better. For one chemical group, *N*_*U*_, *N*_CC_, and *N*_SC_ denoted the number of “*U*,” the number of “CC,” and the number of “SC.” Values in the bracket denoted ratios of *N*_*U*_, *N*_SC_, and *N*_CC_ to total number of chemicals in specific chemical group.

**Table 3:** Overview of the analytical protocols with target chemicals, extraction methods, cleanup methods, and instrument analysis for marine and freshwater fishes.

Fishes	Target chemicals	Extraction methods	Cleanup methods	Instrument analysis	Reference
Seven marine fishes	PBDEs	LLE	GPC + SPE	GC/MS NCI-SIM	[16]
Chub	PCBs, OCPs	PLE	GPC + sulphuric acids	GC/ECD	[17]
Marine fish	PAHs	SE	CC	GC/MS EI-SIM	[18]
Blue mussel, salmon	PAHs	PLE	GPC + SC	GC/MS EI-SIM	[19]
Fifteen carps	PBDEs	PLE	CC	GC/MS EI-SIM	[20]
Atlantic salmon	PBDEs, PCBs	SE	CC (acidic silica gel)	GC/MS ECNI-SIM	[21]
Freshwater fish	PBDEs, PCBs	SE, PLE, MAE	CC (acidic silica gel)	GC/MS EI-SIM	[22]
Trout, salmon	PAHs, PBDEs, PCBs	LLE, DSPE	SPE	GC/TOFMS EI-SIM	[23]
Freshwater fish	OCPs	PLE, SE	CC	GC-ECD	[24]
Marine fish	PCBs, OCPs	PLE	GPC + CC	GC/MS NCI-SIM	[25]
Grass carp	PAHs, OCPs, PBDEs, PCBs	MAE	GPC + SC/CC	GC/MS EI/NCI-SIM	The present study

Notes: LLE, liquid-liquid extraction; PLE, pressurized liquid extraction; SE, Soxhlet extraction; DSPE, dispersive solid phase extraction; ECD, electrical conductivity detector; ECNI, electron capture negative ionization; NCI, negative chemical ionization; EI, electron impact; SIM, selective ion monitoring mode.
