# Supplementary material for: Optimized Multiresidue Analysis of Organic Contaminants of Priority Concern in a Daily Consumed Fish (Grass Carp)
Source: J Anal Methods Chem. 2017 Mar 1;2017:9294024. doi: 10.1155/2017/9294024 (PMC5350539; doi:10.1155/2017/9294024)
Supplement: Supplementary file 1 — The supplemental materials with 30 pages include the instrument conditions of GC/MS matrix in Text S1, fragment ions for identification/quantification plus instrument limit of detection (LOD) for target chemicals in Table S1–S4, ANOVA and IST analysis of extracted target chemicals in blank and spiked fish by EA/CH (1:1), DCM/HEX (4:1), and HEX/ACE (1:1) in Table S5–S7, recoveries (RV), relative standard deviation (RSD), quality judgment (QJ) of target chemicals in spiked fish meat extracted by EA/CH (1:1), DCM/HEX (4:1), and HEX/ACE (1:1) in Table S8, GC/MS abundance of target chemicals at every fraction of GPC from the 10th min in Table S9, ANOVA and IST analysis of recoveries of target chemicals in spiked fish meat cleaned up by SPE cartridge (SC) and chromatography column (CC) in Table S10, recoveries of target chemicals at each SPE eluate when using DCM, DCM/HEX (1:1), HEX in Figures S1–S4, and recoveries with their standard deviation and their relative standard deviation of target chemicals in spiked fish meat cleaned up by SPE cartridge and chromatography column. [file 9294024.f1.pdf]

Supplemental materials for

Optimized multi-residue analysis of organic contaminants of priority concern in a daily consumed fish (grass carp)

Wei He[a], Yanru Chen[a], Chen Yang[a], Wenxiu Liu[a], Xiangzhen Kong[a], Ning Qin[b], Qishuang He[c], Fuliu Xu[a,]*

[a] MOE Laboratory for Earth Surface Process, College of Urban & Environmental Sciences, Peking University, Beijing 100871, China

[b] State Key Laboratory of Environmental Criteria and Risk Assessment, Chinese Research Academy of Environmental Sciences, Beijing 100012, China

[c] Beijing Municipal Key Laboratory of Agriculture Environment Monitoring, Beijing, 100097, China.

*Correspondence author: Tel./Fax: +86 – 10 – 62751177, Email: xufl@urban.pku.edu.cn

The supplemental materials with 30 pages include the instrument conditions of GC/MS matrix in Text S1, fragment ions for identification/quantification plus instrument limit of detection (LOD) for target chemicals in Table S1-S4, ANOVA and IST analysis of extracted target chemicals in blank and spiked fish by EA/CH (1:1), DCM/HEX (4:1), and HEX/ACE(1:1) in Table S5-S7, recoveries (RV), relative standard deviation (RSD), quality judgment (QJ) of target chemicals in spiked fish meat extracted by EA/CH (1:1), DCM/HEX (4:1), and HEX/ACE(1:1) in Table S8, GC/MS abundance of target chemicals at every fraction of GPC from the 10th min in Table S9, ANOVA and IST analysis of recoveries of target chemicals in spiked fish meat cleaned up by SPE cartridge (SC) and chromatography column (CC) in Table S10, recoveries of target chemicals at each SPE eluate

when using DCM, DCM/HEX (1:1), HEX in Fig. S1-S4, and recoveries with their standard deviation and their relative standard deviation of target chemicals in spiked fish meat cleaned up by SPE cartridge and chromatography column.

Text 1 The instrument conditions of GC/MS matrix

1. Analysis of PAHs

Instrument Agilent 6890GC-5973 inert MSD with electron impact (EI) source

Carrier gas helium purity ≥ 99.999%, constant flow 1 mL min^{-1}

Injection splitless injection at 280℃, 1 μL

Column DB-5 MS 30 m, 0.250 mm inner diameter, 0.25 μm film thickness

Oven program 2 min at 100℃, then 5℃min^{-1} to 305℃, then 10 min at 305℃

MS conditions transfer line 300℃, quadrupole 150℃, ion source 246℃

2. Analysis of OCPs

Instrument Agilent 7890GC-5975C MSD with negative chemical ionization (NCI) source

Carrier gas helium purity ≥ 99.999%, constant flow 1 mL min^{-1}

Injection splitless injection at 220℃, 1 μL

Column DB-5MS 30 m, 0.250 mm inner diameter, 0.25 μm film thickness

Oven program 2 min at 50℃, then 10℃min^{-1} to 150℃, then 3℃min^{-1} to 240℃, then 5 min at 240℃, then 10℃min^{-1} to 300℃, then 5 min at 300℃

MS conditions transfer line 250℃, quadrupole 150℃, ion source 200℃

3. Analysis of PBDEs

Instrument Agilent 7890GC-5975C MSD with NCI source

Carrier gas helium purity ≥ 99.999%, constant flow 2 mL min^{-1}

Injection   splitless injection at 265℃, 1 μL

Column   DB-5MS 15 m, 0.250 mm inner diameter, 0.10 μm film thickness

Oven program   5 min at 110℃, then 20℃min^{-1} to 200℃, then 5 min at 200℃, then 10℃min^{-1} to 280℃, then 10 min at 280℃, then 20℃min^{-1} to 305℃, then 10 min at 305℃

MS conditions   transfer line 250℃, quadrupole 150℃, ion source 230℃

4. Analysis of PCBs

Instrument   Varian CP3800-320MS GC-MSD/MSD with EI source

Carrier gas   helium purity ≥ 99.999%, constant flow 1 mL min^{-1}

Injection   splitless injection at 220℃, 1 μL

Column   DB-5MS 30 m, 0.250 mm inner diameter, 0.25 μm film thickness

Oven program   2 min at 100℃, then 5℃min^{-1} to 200℃, then 3℃min^{-1} to 270℃, then 5 min at 270℃, then 10℃min^{-1} to 300℃, then 5 min at 300℃

MS conditions   ion source 230℃

5. Full scan of spiked samples

Instrument   Agilent 6890GC-5973 inert MSD with electron impact (EI) source

Carrier gas   helium purity ≥ 99.999%, constant flow 1 mL min^{-1}

Injection   splitless injection at 220℃, 1 μL

Column   DB-5 MS 30 m, 0.250 mm inner diameter, 0.25 μm film thickness

Oven program   2 min at 100℃, then 10℃min^{-1} to 150℃, then 20℃min^{-1} to 300℃, then 10 min at 300℃

MS conditions   transfer line 280℃, quadrupole 150℃, ion source 246℃

Scanning mass range 50 – 600

Table S1 Fragment ions for identification/quantification plus instrument limit of detection (LOD) and method limit of quantification (LOQ) of PAHs

Analyte (abbreviation)	Item No.	Retention time	*m/z* Identification	Quantification	LOD ng mL^{-1}	LOQ ng g^{-1}
Naphthalene-d_8 (PAH-IS1)	Z-014J-4	8.763	136.0	136.0		
Naphthalene (Nap)	Z-014G	8.763	128.0	128.0	0.5	0.08
2-Fluorobiphenyl (PAH-SS1)	M-625-09	13.153	172.0	172.0	0.5	0.08
Acenaphthylene (Acy)	Z-014G	15.269	152.0	152.0	0.5	0.08
Acenaphthene-d_{10} (PAH-IS2)	M-625-02	15.934	164.0	164.0		
Acenaphthene (Ace)	Z-014G	16.070	153.0, 154.0	153.0	1.0	0.17
Fluorene (Fle)	Z-014G	18.448	166.0	166.0	0.5	0.08
Anthracene-d_{10} (PAH-IS3)	Z-014J-2	22.988	188.0	188.0		
Phenanthrene (Phe)	Z-014G	22.833	178.0	178.0	0.5	0.08
Anthracene (Ant)	Z-014G	23.072	178.0	178.0	0.5	0.08
Fluoranthene (Flu)	Z-014G	28.394	202.0	202.0	0.5	0.08
Pyrene (Pyr)	Z-014G	29.381	202.0	202.0	0.5	0.08
p-Terphenyl-d_{14} (PAH-SS2)	M-525-FS-2	30.586	244.0	244.0	0.5	0.08
Retene (Ret)	Dr. E	31.090	219.0, 234.0	219.0	2.0	0.33
Benzo[c]phenanthrene (BcP)	H-244S	34.136	228.0	228.0	1.0	0.17
Cyclopenta[c,d]pyrene (CcdP)	H-242S	34.988	226.0	226.0	1.0	0.17
Benz[a]anthracene (BaA)	Z-014G	35.135	228.0	228.0	1.0	0.17
Chrysene-d_{12} (PAH-IS4)	Z-014J-4	35.135	240.0	240.0		
Chrysene (Chr)	Z-014G	35.262	228.0	228.0	1.0	0.17
Benzo[b]fluoranthene (BbF)	Z-014G	39.870	252.0	252.0	1.0	0.17
Benzo[k]fluoranthene (BkF)	Z-014G	39.999	252.0	252.0	1.0	0.17
Benzo[e]pyrene (BeP)	H-112S	41.111	252.0	252.0	1.0	0.17
Benzo[a]pyrene (BaP)	Z-014G	41.361	252.0	252.0	1.0	0.17
Perylene-d_{12} (PAH-IS5)	Z-014J-5	41.666	264.0	264.0		
Perylene (Per)	H-121S	41.790	252.0	252.0	1.0	0.17
Indeno[1,2,3-cd]pyrene (IcdP)	Z-014G	47.061	276.0	276.0	1.0	0.17
Dibenz[a,h]anthracene (DahA)	Z-014G	47.270	278.0	278.0	1.0	0.17
Benzo[g,h,i]perylene (BghiP)	Z-014G	48.175	276.0	276.0	1.0	0.17
Anthanthrene (Antt)	H-109S	48.811	276.0	276.0	2.0	0.33
Dibenzo[a,l]pyrene (DiBalP)	H-179S	53.351	302.0	302.0	2.0	0.33
Dibenzo[a,e]fluoranthene (DiBaeF)	H-247S	53.572	302.0	302.0	2.0	0.33
Coronene (Cor)	H-116S	54.970	300.0	300.0	1.0	0.17
di-benzo[a,e]pyrene (DiBaeP)	H-138S	55.098	302.0	302.0	2.0	0.33
di-benzo[a,i]pyrene (DiBaiP)	H-178S	55.794	302.0	302.0	2.0	0.33
di-benzo[a,h]pyrene (DiBahP)	H-177S	56.154	302.0	302.0	2.0	0.33

Note: only retene was supplied by Dr. Ehrenstorfer GmbH, Germany, others were supplied by AccuStandard Inc., NY, US with item number.

Table S2 Fragment ions for identification/quantification plus instrument limit of detection (LOD) and method limit of quantification (LOQ) of OCPs

Analyte (abbreviation)	Item No.	Retention time	*m/z* Identification	Quantification	LOD ng mL^{-1}	LOQ ng g^{-1}
Pentachloronitrobenzene (OCP-IS)	M-508.1-IS	20.057	248.9	248.9		
1-Bromo-2-nitrobenzene (OCP-SS)	M-8081-IS	12.064	79.1, 81.1	81.0	0.2	0.03
α-HCH	AE-00010	18.794	71.0, 255.0	71.0	0.5	0.08
Hexachlorbenzene (HCB)	AE-00010	19.106	283.9, 285.8	283.9	<0.05	0.01
β-HCH	AE-00010	20.075	71.0, 255.0	71.0	0.5	0.08
γ-HCH	AE-00010	20.320	71.0, 255.0	71.0	0.5	0.08
δ-HCH	AE-00010	21.236	71.0, 255.0	71.0	0.5	0.08
Heptachlor (HC)	AE-00010	23.877	237.0, 266.0	266.0	0.5	0.08
Aldrin (Ald)	AE-00010	25.661	35.0, 237.0	237.0	0.5	0.08
Isodrin (ID)	AE-00010	27.202	35.0, 237.0	35.0	0.2	0.03
cis-Heptachlorepoxide (cHCP)	AE-00010	27.958	315.0, 388.0	388.0	5.0	0.83
oxy-Chlordane (oCd)	AE-00010	27.958	350.0, 424.0	424.0	5.0	0.83
trans-Heptachlorepoxide (tHCP)	AE-00010	28.147	315.0, 388.0	388.0	5.0	0.83
trans-Chlordane (tCd)	AE-00010	29.219	300.0, 410.0	410.0	0.5	0.08
o,p'-DDE	AE-00010	29.349	35.0, 246.0	246.0	1.0	0.17
Endosulfan I (ES I)	AE-00010	29.844	35.0, 406.0	406.0	0.5	0.08
cis-Chlordane (cCd)	AE-00010	30.076	300.0, 410.0	410.0	1.0	0.17
Dieldrin (Dld)	AE-00010	31.315	35.0, 237.0	35.0	0.5	0.08
p,p'-DDE	AE-00010	31.526	35.0, 37.0	35.0	0.5	0.08
o,p'-DDD	AE-00010	31.961	35.0, 248.0	35.0	0.5	0.08
Endrin (Ed)	AE-00010	31.484	35.0, 272.0	35.0	5.0	0.83
Endosulfan II (ES II)	AE-00010	33.058	370.0, 406.0	406.0	0.5	0.08
p,p'-DDD	AE-00010	33.881	35.0, 248.0	35.0	5.0	0.83
o,p'-DDT	AE-00010	34.005	35.0, 248.0	35.0	5.0	0.83
p,p'-DDT	AE-00010	35.973	35.0, 71.0	35.0	0.5	0.08
Methoxychlor (MOC)	AE-00010	39.992	396.0, 398.0	396.0	0.5	0.08
Mirex	AE-00010	41.020	35.0, 368.0	368.0	1.0	0.17

Note: all chemicals were supplied by AccuStandard Inc., NY, US with item number.

Table S3 Fragment ions for identification/quantification plus instrument limit of detection (LOD) and method limit of quantification (LOQ) of PBDEs

Analyte (abbreviation)	Item No.	Retention time	*m/z*		LOD ng mL^{-1}	LOQ ng g^{-1}
			Identification	Quantification		
2, 2', 3, 3', 4, 5, 5', 6, 6'-NonaCB	EC-1419-1.2	16.528	473.8, 475.8	475.8		
$^{13}C_{12}$(PBDE-IS)						
2, 2', 4-TriBDE (PBDE 17)	BDE-COC	10.726	79.1, 81.1	79.1	0.5	0.08
2, 4, 4'-TriBDE (PBDE 28)	BDE-COC	11.168	79.1, 81.1	81.1	0.5	0.08
2, 2', 3, 4, 5, 5'-HexaCB	EC-1418-1.2	11.754	372.0, 374.0	372.0	0.5	
$^{13}C_{12}$(PBDE-SS)						0.08
2, 3', 4', 6-TetraBDE (PBDE 71)	BDE-COC	13.892	79.1, 81.1	79.1	0.5	0.08
2, 2', 4, 4'-TetraBDE (PBDE 47)	BDE-COC	14.794	79.1, 81.1	81.1	0.5	0.08
2, 3', 4, 4'-TetraBDE (PBDE 66)	BDE-COC	15.435	79.1, 81.1	79.1	0.5	0.08
2, 2', 4, 4', 6-PentaBDE (PBDE 100)	BDE-COC	17.445	79.1, 81.1	79.1	0.5	0.08
2, 2', 4, 4', 5-PentaBDE (PBDE 99)	BDE-COC	18.143	79.1, 81.1	79.1	0.5	0.08
2, 2', 3, 4, 4'-PentaBDE (PBDE 85)	BDE-COC	19.320	79.1, 81.1	81.1	0.5	0.08
2, 2', 4, 4', 5, 6'-HexaBDE (PBDE 154)	BDE-COC	19.550	79.1, 81.1	79.1	0.5	0.08
2, 2', 4, 4', 5, 5'-HexaBDE (PBDE 153)	BDE-COC	20.346	79.1, 81.1	81.1	0.5	0.08
2, 2', 3, 4, 4', 5'-HexaBDE (PBDE 138)	BDE-COC	21.304	79.1, 81.1	79.1	0.5	0.08
2, 2', 3, 4, 4', 5', 6-HeptaBDE (PBDE 183)	BDE-COC	22.147	79.1, 81.1	81.1	0.5	0.08
2, 3, 3', 4, 4', 5, 6-HeptaBDE (PBDE 190)	BDE-COC	23.255	79.1, 81.1	81.1	0.5	0.08
DecaBDE (PBDE 209)	BDE-COC	30.473	486.6, 488.6	487.0	2.5	0.42

Note: IS and SS were supplied by Cambridge Isotope Laboratorie, Inc., US, others were supplied by AccuStandard Inc., NY, US with item number. PCBs and PBDEs had the same IS and SS.

Table S4 Fragment ions for identification/quantification plus instrument limit of detection (LOD) and method limit of quantification (LOQ) of PCBs (MS1 and MS2 offer precursor ions and product ions, respectively)

Analyte (abbreviation)	Item No.	Retention time	*m/z*			LOD ng mL^{-1}	LOQ ng g^{-1}
			Identification		Quantification		
2, 2', 5-TriCB (PCB 18)	C-SCA-SET	18.684	258.0>186.0	186.0>151.0	186.0>151.0	0.5	0.08
2, 2', 3, 5'-TetraCB (PCB 44)	C-SCA-SET	21.963	220.0>150.0	291.9>219.8	291.9>219.8	1	0.17
2, 2', 4, 5'-TetraCB (PCB 49)	C-SCA-SET	22.106	220.0>150.0	291.9>219.8	291.9>219.8	0.5	0.08
2, 2', 5, 5'-TetraCB (PCB 52)	C-SCA-SET	22.701	220.0>149.8	291.9>219.6	220.0>149.8	0.5	0.08
3, 4, 4'-TriCB (PCB 37)	C-SCA-SET	23.044	256.0>186.0	186.0>150.1	186.0>150.1	0.5	0.08
2, 3', 4', 5-TetraCB (PCB 70)	C-SCA-SET	24.278	291.9>222.0	220.0>150.0	291.9>222.0	0.5	0.08
2, 4, 4', 5-TetraCB (PCB 74)	C-SCA-SET	24.438	291.9>222.0	220.0>150.0	291.9>222.0	0.5	0.08
2, 2', 3, 4, 5'-PentaCB (PCB 87)	C-SCA-SET	25.553	325.8>255.9	254.0>184.0	325.8>255.9	0.5	0.08
2, 2', 4, 4', 5-PentaCB (PCB 99)	C-SCA-SET	25.739	325.8>255.9	254.0>184.0	325.8>255.9	0.5	0.08
2, 3, 4, 4', 5-PentaCB (PCB 114)	C-SCA-SET	25.975	325.8>255.9	254.0>184.0	325.8>255.9	0.5	0.08
2, 2', 4, 5, 5'-PentaCB (PCB 101)	C-SCA-SET	26.704	325.8>255.9	253.9>184.0	325.8>255.9	2.5	0.42
3, 4, 4', 5-TetraCB (PCB 81)	C-SCA-SET	26.794	291.9>222.0		291.9>222.0	2.5	0.42
3, 3', 4, 4'-TetraCB (PCB 77)	C-SCA-SET	27.273	290.0>220.0		290.0>220.0	1	0.17
2, 2', 3, 5, 5', 6-HexaCB (PCB 151)	C-SCA-SET	27.628	290.0>220.0	359.8>290.0	359.8>290.0	0.5	0.08
2, 3', 4, 4', 6-PentaCB (PCB 119)	C-SCA-SET	28.237	325.9>255.8	254.0>184.0	325.9>255.8	1	0.17
2, 3, 3', 4, 4'-PentaCB (PCB 105)	C-SCA-SET	28.431	325.9>255.8	254.0>184.0	325.9>255.8	0.5	0.08
2', 3, 4, 4', 5-PentaCB (PCB 123)	C-SCA-SET	28.961	325.9>255.8	254.0>184.0	325.9>255.8	1	0.17
2, 2', 4, 4', 5, 5'-HexaCB (PCB 153)	C-SCA-SET	29.461	288.0>218.0	359.9>290.0	359.9>290.0	0.5	0.08
2, 3, 3', 4, 4', 6-HexaCB (PCB 157)	C-SCA-SET	29.461	288.0>218.0	359.9>290.0	359.9>290.0	0.5	0.08
2, 3', 4, 4', 5-PentaCB (PCB 118)	C-SCA-SET	29.613	325.9>256.0		325.9>256.0	2.5	0.42
2, 2', 3, 4, 5, 5'-HexaCB $^{13}C_{12}$(PCB-SS)	EC-1418-1.2	30.051	372.0>302.0		372.0>302.0	0.5	0.08
2, 2', 3, 3', 4, 4'-HexaCB (PCB 128)	C-SCA-SET	30.792	288.0>218.0	359.9>290.0	359.9>290.0	0.5	0.08
2, 3, 3', 4, 4', 5'-HexaCB (PCB 158)	C-SCA-SET	30.792	288.0>218.0	359.9>290.0	359.9>290.0	0.5	0.08
3, 3', 4, 4', 5-PentaCB (PCB 126)	C-SCA-SET	31.405	326.0>256.0		326.0>256.0	2.5	0.42
2, 2', 3, 4, 4', 5, 5'-HeptaCB (PCB 180)	C-SCA-SET	31.485	395.7>325.6		395.7>325.6	2.5	0.42
2, 2', 3, 3', 4', 5, 6-HeptaCB (PCB 177)	C-SCA-SET	31.758	395.7>325.6		395.7>325.6	2.5	0.42
2, 2', 3, 4, 4', 5'-HexaCB (PCB 138)	C-SCA-SET	32.011	359.8>290.0		359.8>290.0	2.5	0.42
2, 3', 4, 4', 5, 5'-HexaCB (PCB 167)	C-SCA-SET	32.266	359.8>290.0		359.8>290.0	2.5	0.42
2, 2', 3, 4', 5, 5', 6-HeptaCB (PCB 187)	C-SCA-SET	32.890	393.8>323.8	395.7>323.9	395.7>323.9	1	0.17
2, 3, 3', 4, 4', 5-HexaCB (PCB 156)	C-SCA-SET	33.335	359.8>290.0		359.8>290.0	2.5	0.42
2, 3', 4, 4', 5', 6-HexaCB (PCB 168)	C-SCA-SET	33.577	359.8>290.0		359.8>290.0	2.5	0.42
2, 2', 3, 4, 4', 5', 6-HeptaCB (PCB 183)	C-SCA-SET	34.183	393.8>323.8	395.7>323.9	393.8>323.8	2.5	0.42
3, 3', 4, 4', 5, 5'-HexaCB (PCB 169)	C-SCA-SET	35.367	360.0>290.0	361.8>292.0	361.8>292.0	5	0.83
2, 2', 3, 3', 4, 4', 5-HeptaCB (PCB 170)	C-SCA-SET	35.579	395.9>323.7		395.9>323.7	5	0.83
2, 2', 3, 3', 4, 5, 5', 6'-OctaCB (PCB 199)	C-SCA-SET	35.962	360.0>290.0	427.8>357.7	360.0>290.0	5	0.83
2, 3, 3', 4, 4', 5, 5'-HeptaCB (PCB 189)	C-SCA-SET	37.202	393.8>324.0		393.8>324.0	0.5	0.08
2, 2', 3, 3', 4, 5, 5', 6, 6'-NonaCB $^{13}C_{12}$(PCB-IS)	EC-1419-1.2	37.672	475.7>406.0		475.7>406.0		
2, 2', 3, 3', 4, 4', 5, 5'-OctaCB (PCB 194)	C-SCA-SET	38.964	427.7>357.9		427.7>357.9	0.5	0.08

Table S5 ANOVA and IST analysis of extracted target chemicals by EA/CH (1:1), DCM/HEX (4:1), and HEX/ACE (1:1) in blank fish meat

Analytes	Extract solvent (ng / 2 g)			TOHOV	ANOVA	IST (ES1 vs. ES2)			IST (ES1 vs. ES3)			IST (ES2 vs. ES3)		
	ES1	ES2	ES3	p	p	p_L	p_e	p_i	p_L	p_e	p_i	p_L	p_e	p_i
**PAHs**														
Nap	89.91	91.84	99.75	0.258	0.287	0.131	0.616	0.547	0.217	0.261	0.216	0.399	0.266	0.290
PAH-SS1	152.16	152.76	147.35	0.253	0.570	0.695	0.869	0.882	0.328	0.543	0.480	0.184	0.383	0.411
Any	2.95	5.45	6.71	0.004	0.248	0.023	0.382	0.547	0.003	0.196	0.419	0.226	0.263	0.284
Ace	14.63	13.76	15.52	0.304	0.170	0.138	0.280	0.219	0.228	0.404	0.332	0.600	0.109	0.116
Fle	51.32	49.42	52.67	0.560	0.635	0.392	0.542	0.485	0.385	0.754	0.708	0.639	0.415	0.421
Phe	118.99	87.45	116.14	0.090	0.302	0.268	0.058	0.057	0.093	0.918	0.899	0.146	0.250	0.281
Ant	11.22	7.97	10.32	0.102	0.244	0.097	0.098	0.090	0.076	0.664	0.598	0.467	0.262	0.268
Flu	51.28	64.03	53.15	0.045	0.898	0.070	0.759	0.707	0.062	0.902	0.879	0.100	0.746	0.755
Pyr	112.13	108.72	78.05	0.150	0.526	0.202	0.939	0.925	0.473	0.094	0.087	0.147	0.403	0.435
PAH-SS2	275.08	245.13	224.71	0.073	0.089	0.149	0.024	0.114	0.195	0.110	0.081	0.060	0.298	0.347
Baa	6.56	6.15	5.19	0.122	0.845	0.276	0.904	0.891	0.304	0.429	0.533	0.086	0.698	0.710
Chr	6.01	6.00	5.47	0.078	0.962	0.167	0.998	0.998	0.558	0.639	0.684	0.067	0.831	0.839
BbF	6.66	4.81	4.57	0.001	0.292	0.010	0.329	0.524	0.000	0.261	0.487	0.247	0.614	0.627
BkF	2.34	2.70	1.10	0.022	0.425	0.172	0.843	0.814	0.000	0.093	0.317	0.020	0.266	0.325
BaP	12.35	22.12	18.78	0.025	0.654	0.063	0.501	0.428	0.103	0.123	0.097	0.041	0.759	0.771
IcdP	1.30	1.06	1.02	0.035	0.750	0.003	0.497	0.658	0.860	0.601	0.631	0.044	0.904	0.909
DiahA	0.00	0.00	0.00	-	-	-	-	-	-	-	-	-	-	-
BghiP	2.86	2.29	2.52	0.556	0.359	0.249	0.218	0.337	0.491	0.456	0.527	0.744	0.462	0.463
**OCPs**														
OCP-SS	81.83	75.90	82.61	0.146	0.686	0.514	0.342	0.348	0.219	0.939	0.940	0.103	0.496	0.517
α-HCH	5.86	2.98	2.99	0.297	0.116	0.151	0.078	0.103	0.207	0.073	0.096	0.894	0.992	0.992
**HCB**	4.83	4.55	4.83	0.061	**0.044**	0.102	0.074	0.120	0.725	1.000	1.000	0.080	0.071	0.123
β-HCH	2.56	1.98	1.95	0.527	0.369	0.586	0.208	0.213	0.280	0.249	0.263	0.591	0.960	0.960
γ-HCH	1.08	0.55	0.76	0.051	0.125	0.252	0.113	0.128	0.039	0.237	0.289	0.079	0.217	0.255
δ-HCH	0.31	0.28	0.11	0.365	0.486	0.249	0.891	0.895	0.555	0.375	0.385	0.188	0.220	0.239

HC	0.00	0.00	0.00	-	-	-	-	-	-	-	-	-	-	-
Ald	0.00	0.00	0.00	-	-	-	-	-	-	-	-	-	-	-
ID	0.16	0.25	0.00	0.064	0.311	0.311	0.658	0.666	0.039	0.127	0.195	0.053	0.201	0.266
cHCP	0.00	0.00	0.00	-	-	-	-	-	-	-	-	-	-	-
oCd	0.00	0.00	0.00	-	-	-	-	-	-	-	-	-	-	-
tHCP	0.00	0.00	0.00	-	-	-	-	-	-	-	-	-	-	-
tCd	0.00	0.00	0.00	-	-	-	-	-	-	-	-	-	-	-
o,p'-DDE	0.00	0.00	0.00	-	-	-	-	-	-	-	-	-	-	-
ES I	1.80	1.11	1.61	0.018	0.389	0.046	0.297	0.341	0.558	0.359	0.362	0.036	0.429	0.466
cCd	0.00	0.00	0.00	-	-	-	-	-	-	-	-	-	-	-
Dld	0.00	0.00	0.00	-	-	-	-	-	-	-	-	-	-	-
p,p'-DDE	12.37	12.18	12.52	0.436	0.812	0.232	0.727	0.732	0.464	0.759	0.761	0.600	0.593	0.594
o,p'-DDD	2.09	2.08	2.15	0.080	0.918	0.289	0.967	0.968	0.039	0.767	0.779	0.179	0.603	0.620
Ed	0.00	0.00	0.00	-	-	-	-	-	-	-	-	-	-	-
ES II	0.42	11.45	0.27	0.004	0.414	0.017	0.372	0.421	0.817	0.472	0.472	0.017	0.366	0.415
p,p'-DDD	9.25	9.60	9.20	0.194	0.786	0.414	0.482	0.491	0.097	0.941	0.943	0.330	0.607	0.612
o,p'-DDT	1.57	1.33	1.29	0.680	0.554	0.496	0.396	0.401	0.418	0.329	0.335	0.939	0.889	0.889
p,p'-DDT	2.20	0.86	0.94	0.146	0.296	0.056	0.213	0.258	0.365	0.111	0.139	0.312	0.947	0.947
MOC	0.00	0.00	0.00	-	-	-	-	-	-	-	-	-	-	-
Mirex	0.00	0.00	0.00	-	-	-	-	-	-	-	-	-	-	-
**PBDEs**														
PBDE 17	0.00	0.00	0.00	-	-	-	-	-	-	-	-	-	-	-
PBDE 28	0.25	0.22	0.18	0.100	0.571	0.090	0.634	0.653	0.409	0.116	0.129	0.174	0.680	0.690
PBDE-SS	103.93	100.33	100.23	0.425	0.112	0.459	0.125	0.132	0.235	0.098	0.119	0.630	0.946	0.947
PBDE 71	0.00	0.00	0.00	-	-	-	-	-	-	-	-	-	-	-
PBDE 47	0.63	0.50	0.48	0.533	0.244	0.305	0.220	0.234	0.670	0.109	0.112	0.531	0.815	0.816
PBDE 66	0.00	0.00	0.00	-	-	-	-	-	-	-	-	-	-	-
PBDE 100	0.00	0.00	0.00	-	-	-	-	-	-	-	-	-	-	-
PBDE 99	0.00	0.00	0.00	-	-	-	-	-	-	-	-	-	-	-
PBDE 85	0.00	0.00	0.00	-	-	-	-	-	-	-	-	-	-	-

PBDE 154	0.09	0.09	0.10	0.169	0.847	0.823	0.882	0.882	0.145	0.539	0.566	0.057	0.692	0.708
PBDE 153	0.00	0.00	0.00	-	-	-	-	-	-	-	-	-	-	-
PBDE 138	0.66	0.75	0.52	0.079	0.559	0.201	0.727	0.732	0.219	0.340	0.359	0.061	0.361	0.398
PBDE 183	0.00	0.00	0.00	-	-	-	-	-	-	-	-	-	-	-
PBDE 190	0.00	0.00	0.00	-	-	-	-	-	-	-	-	-	-	-
**PBDE 209**	2.26	4.96	3.01	0.070	0.092	0.110	0.099	0.165	**0.423**	**0.002**	**0.002**	0.104	0.197	0.261
**PCBs**														
PCB 18	0.15	0.21	0.23	0.831	0.097	0.731	0.114	0.116	0.595	0.078	0.064	0.764	0.448	0.45
PCB 44	0.00	0.00	0.07	0.011	0.507	-	-	-	0.053	0.495	0.423	0.016	0.374	0.423
PCB 49	0.15	0.29	0.10	0.131	0.352	0.235	0.454	0.547	0.056	0.727	0.797	0.361	0.125	0.135
**PCB 52**	0.00	0.07	0.10	0.025	0.414	0.053	0.495	0.423	**0.118**	**0.022**	**0.03**	0.048	0.654	0.671
PCB 37	0.00	0.13	0.08	0.054	0.321	0.056	0.220	0.184	0.056	0.22	0.184	0.276	0.556	0.562
PCB 70	0.00	0.00	0.04	0.011	0.507	-	-	-	0.053	0.495	0.423	0.016	0.374	0.423
PCB 74	0.00	0.10	0.04	0.093	0.363	0.085	0.229	0.191	0.053	0.495	0.423	0.619	0.41	0.413
**PCB 87**	0.00	0.05	0.16	0.031	0.079	0.053	0.495	0.423	**0.058**	**0.009**	**0.016**	0.082	0.134	0.173
**PCB 99**	0.00	0.04	0.12	0.028	0.087	0.053	0.495	0.423	**0.193**	**0.004**	**0.009**	0.052	0.149	0.199
PCB 114	0.00	0.00	0.00	-	-	-	-	-	-	-	-	-	-	-
PCB 101	0.00	0.07	0.05	0.076	0.351	0.063	0.222	0.186	0.067	0.223	0.187	0.507	0.717	0.718
PCB 81	0.00	0.00	0.00	-	-	-	-	-	-	-	-	-	-	-
PCB 77	0.00	0.07	0.07	0.044	0.641	0.053	0.495	0.423	0.078	0.226	0.189	0.146	0.977	0.977
PCB 151	0.00	0.00	0.00	-	-	-	-	-	-	-	-	-	-	-
PCB 119	0.00	0.00	0.00	-	-	-	-	-	-	-	-	-	-	-
PCB 105	0.00	0.10	0.12	0.156	0.382	0.118	0.239	0.199	0.101	0.233	0.195	0.704	0.803	0.804
PCB 123	0.00	0.00	0.00	-	-	-	-	-	-	-	-	-	-	-
PCB 153/157	0.00	0.04	0.11	0.081	0.355	0.053	0.495	0.423	0.074	0.225	0.188	0.58	0.401	0.404
PCB 118	0.00	0.00	0.00	-	-	-	-	-	-	-	-	-	-	-
PCB-SS	44.95	59.82	64.78	0.001	0.005	0.000	0.030	0.210	0.017	0.019	0.138	0.021	0.028	0.077
PCB 128/158	0.07	0.04	0.14	0.020	0.265	0.760	0.797	0.815	0.001	0.236	0.462	0.03	0.103	0.163
PCB 126	0.00	0.00	0.00	-	-	-	-	-	-	-	-	-	-	-
PCB 180	0.00	0.00	0.00	-	-	-	-	-	-	-	-	-	-	-

PCB 177	0.00	0.00	0.00	-	-	-	-	-	-	-	-	-	-	-
PCB 138	0.00	0.00	0.00	-	-	-	-	-	-	-	-	-	-	-
PCB 167	0.00	0.00	0.00	-	-	-	-	-	-	-	-	-	-	-
PCB 187	0.00	0.00	0.00	-	-	-	-	-	-	-	-	-	-	-
PCB 156	0.00	0.00	0.00	-	-	-	-	-	-	-	-	-	-	-
PCB 168	0.00	0.00	0.00	-	-	-	-	-	-	-	-	-	-	-
PCB 183	0.00	0.00	0.00	-	-	-	-	-	-	-	-	-	-	-
PCB 169	0.00	0.00	0.00	-	-	-	-	-	-	-	-	-	-	-
PCB 170	0.00	0.00	0.00	-	-	-	-	-	-	-	-	-	-	-
PCB 199	0.00	0.00	0.00	-	-	-	-	-	-	-	-	-	-	-
PCB 189	0.00	0.00	0.00	-	-	-	-	-	-	-	-	-	-	-
PCB 194	0.00	0.00	0.00	-	-	-	-	-	-	-	-	-	-	-

Notes: ES1 – EA/CH (1:1), ES2 – DCM/HEX (4:1), ES3 – HEX/ACE(1:1), TOHOV –Test of homogeneity of variances, ANOVA – analysis of variances, IST –Independent sample test, p_L – Significance of the Levene' test, p_e – Significance of IST when variances are equal, p_i – Significance of IST when variances are inequal, “-“ means the dataset is not suitable to be tested.

Table S6 ANOVA and IST analysis of extracted target chemicals by EA/CH (1:1), DCM/HEX (4:1), and HEX/ACE (1:1) in spiked fish meat

Analytes	Extract solvent (ng / 2 g)			TOHOV	ANOVA	IST (ES1 vs. ES2)			IST (ES1 vs. ES3)			IST (ES2 vs. ES3)		
	ES1	ES2	ES3	*p*	*p*	p_L	p_e	p_i	p_L	p_e	p_i	p_L	p_e	p_i
**PAHs**														
Nap	221.74	243.18	232.58	0.092	0.240	0.076	0.110	0.099	0.066	0.380	0.315	0.781	0.390	0.390
PAH-SS1	160.47	153.66	149.58	0.098	0.738	0.894	0.356	0.364	0.155	0.560	0.489	0.095	0.774	0.783
Any	125.24	131.39	134.66	0.171	0.224	0.912	0.078	0.101	0.229	0.198	0.151	0.147	0.512	0.534
Ace	150.62	154.67	151.62	0.062	0.332	0.190	0.312	0.453	0.003	0.748	0.830	0.340	0.135	0.171
Fle	210.31	218.77	211.65	0.177	0.865	0.187	0.712	0.659	0.621	0.908	0.904	0.177	0.700	0.709
Phe	245.97	277.69	261.64	0.028	0.872	0.086	0.707	0.648	0.960	0.426	0.454	0.033	0.802	0.812
Ant	153.72	155.39	152.05	0.048	0.895	0.138	0.878	0.851	0.308	0.545	0.629	0.045	0.688	0.706
Flu	237.78	235.67	230.85	0.039	0.970	0.174	0.960	0.952	0.049	0.624	0.728	0.033	0.875	0.881
Pyr	242.91	251.36	238.03	0.661	0.792	0.446	0.734	0.715	0.672	0.814	0.814	0.602	0.540	0.542
PAH-SS2	181.59	174.35	168.09	0.015	0.223	0.022	0.427	0.584	0.017	0.187	0.379	0.921	0.172	0.172
Baa	205.64	206.10	204.22	0.097	0.887	0.057	0.813	0.861	0.249	0.815	0.780	0.074	0.679	0.698
Chr	96.60	98.41	98.65	0.125	0.812	0.757	0.380	0.360	0.149	0.641	0.577	0.124	0.944	0.946
BbF	195.90	196.19	187.34	0.071	0.403	0.999	0.977	0.978	0.004	0.244	0.455	0.070	0.211	0.262
BkF	166.39	164.46	156.81	0.029	0.285	0.791	0.816	0.820	0.001	0.119	0.344	0.030	0.192	0.252
**BaP**	202.35	211.35	200.74	0.017	0.008	0.046	0.048	0.183	0.010	0.577	0.701	**0.378**	**0.002**	**0.003**
IcdP	142.41	142.56	140.10	0.500	0.715	0.246	0.967	0.973	0.949	0.606	0.631	0.382	0.456	0.461
DiahA	66.92	68.02	66.47	0.985	0.832	0.872	0.722	0.730	0.883	0.888	0.889	0.982	0.580	0.580
BghiP	85.44	86.86	85.99	0.465	0.904	0.398	0.617	0.588	0.326	0.885	0.866	0.522	0.795	0.796
**OCPs**														
**OCP-SS**	72.76	66.63	83.76	0.418	0.013	0.740	0.253	0.254	**0.366**	**0.032**	**0.050**	**0.170**	**0.010**	**0.024**
α-HCH	51.44	54.94	51.21	0.053	0.386	0.063	0.343	0.390	0.245	0.855	0.858	0.117	0.331	0.367
HCB	50.60	50.50	50.02	0.591	0.622	0.808	0.856	0.856	0.506	0.431	0.435	0.384	0.495	0.501
β-HCH	43.74	46.11	46.03	0.038	0.579	0.056	0.464	0.502	0.089	0.488	0.519	0.214	0.913	0.915
γ-HCH	45.59	47.00	47.02	0.071	0.452	0.095	0.377	0.410	0.097	0.372	0.404	0.847	0.976	0.976
δ-HCH	39.61	40.12	40.75	0.114	0.879	0.146	0.855	0.860	0.115	0.680	0.693	0.887	0.630	0.631

HC	49.95	50.66	50.83	0.920	0.663	0.795	0.496	0.496	0.891	0.445	0.445	0.713	0.873	0.873
Ald	53.88	53.33	51.95	0.490	0.388	0.679	0.743	0.743	0.457	0.156	0.181	0.225	0.348	0.375
ID	47.91	41.81	46.05	0.009	0.472	0.033	0.355	0.400	0.316	0.183	0.207	0.022	0.503	0.538
cHCP	39.69	46.85	47.50	0.107	0.344	0.251	0.123	0.150	0.062	0.246	0.291	0.269	0.925	0.926
oCd	48.94	50.49	51.09	0.050	0.769	0.573	0.369	0.378	0.097	0.580	0.596	0.049	0.868	0.875
tHCP	42.98	45.46	45.19	0.261	0.467	0.361	0.194	0.204	0.162	0.363	0.386	0.404	0.916	0.917
tCd	45.16	47.75	42.74	0.020	0.375	0.828	0.084	0.084	0.044	0.574	0.599	0.042	0.273	0.323
o,p'-DDE	52.12	52.48	51.87	0.791	0.942	0.765	0.844	0.844	0.588	0.901	0.890	0.553	0.719	0.711
ES I	51.13	51.73	50.81	0.220	0.752	0.217	0.685	0.693	0.183	0.827	0.832	0.986	0.310	0.311
cCd	45.18	45.52	44.52	0.196	0.722	0.459	0.827	0.829	0.107	0.631	0.650	0.217	0.311	0.344
Dld	68.52	68.28	67.28	0.341	0.852	0.542	0.934	0.934	0.206	0.628	0.642	0.289	0.597	0.604
p,p'-DDE	58.12	58.15	55.71	0.457	0.245	0.190	0.983	0.983	0.608	0.229	0.232	0.513	0.122	0.139
o,p'-DDD	47.49	47.61	46.50	0.210	0.691	0.364	0.946	0.947	0.158	0.554	0.582	0.155	0.208	0.245
Ed	54.20	53.43	53.16	0.240	0.785	0.244	0.691	0.704	0.227	0.588	0.608	0.966	0.740	0.740
**ES II**	45.49	44.61	46.71	0.010	0.506	0.031	0.693	0.711	0.026	0.585	0.611	**0.696**	**0.009**	**0.010**
p,p'-DDD	63.35	62.22	61.54	0.814	0.708	0.662	0.650	0.654	0.632	0.474	0.481	0.916	0.719	0.719
**o,p'-DDT**	57.32	58.32	53.50	0.110	0.148	0.081	0.693	0.704	0.225	0.212	0.228	**0.287**	**0.034**	**0.043**
p,p'-DDT	50.80	49.90	48.67	0.047	0.282	0.193	0.572	0.582	0.038	0.180	0.239	0.097	0.157	0.203
MOC	44.87	50.10	46.90	0.232	0.959	0.424	0.824	0.827	0.136	0.922	0.926	0.268	0.796	0.804
Mirex	43.74	43.84	45.00	0.083	0.838	0.269	0.972	0.973	0.038	0.632	0.653	0.219	0.495	0.518
**PBDEs**														
PBDE 17	26.25	27.22	27.20	0.662	0.184	0.490	0.160	0.180	0.863	0.077	0.077	0.546	0.965	0.966
PBDE 28	26.69	27.41	27.44	0.243	0.331	0.653	0.306	0.308	0.241	0.156	0.188	0.104	0.950	0.951
**PBDE-SS**	102.98	106.58	104.77	0.088	0.060	**0.053**	**0.044**	**0.087**	0.186	0.089	0.120	0.272	0.267	0.281
PBDE 71	25.98	26.20	26.46	0.159	0.552	0.828	0.684	0.684	0.082	0.279	0.333	0.100	0.498	0.531
PBDE 47	25.44	25.71	26.08	0.581	0.450	0.562	0.638	0.642	0.396	0.274	0.295	0.621	0.376	0.378
PBDE 66	27.66	27.61	28.23	0.517	0.676	0.633	0.956	0.957	0.323	0.504	0.519	0.430	0.357	0.365
PBDE 100	23.26	23.19	23.73	0.645	0.541	0.681	0.912	0.913	0.410	0.413	0.428	0.575	0.261	0.268
PBDE 99	23.28	22.90	23.82	0.396	0.588	0.326	0.710	0.716	0.318	0.602	0.611	0.928	0.215	0.215
PBDE 85	28.22	27.53	29.12	0.235	0.524	0.147	0.660	0.669	0.224	0.580	0.588	0.975	0.176	0.178

PBDE 154	22.98	22.44	23.45	0.517	0.521	0.218	0.556	0.565	0.526	0.643	0.644	0.718	0.231	0.239
PBDE 153	21.98	21.35	22.57	0.249	0.587	0.119	0.615	0.629	0.415	0.679	0.682	0.401	0.228	0.247
PBDE 138	26.85	25.73	27.09	0.094	0.668	0.050	0.499	0.533	0.602	0.907	0.907	0.077	0.318	0.367
PBDE 183	24.89	23.63	24.53	0.131	0.722	0.089	0.456	0.496	0.700	0.859	0.860	0.056	0.481	0.518
PBDE 190	18.98	17.37	18.28	0.257	0.586	0.157	0.354	0.391	0.530	0.714	0.717	0.204	0.447	0.467
PBDE 209	189.13	165.48	195.69	0.096	0.709	0.034	0.517	0.547	0.821	0.892	0.892	0.092	0.396	0.436
**PCBs**														
PCB 18	25.14	25.71	25.96	0.415	0.681	0.510	0.617	0.621	0.462	0.325	0.331	0.260	0.810	0.814
PCB 44	28.52	29.03	29.75	0.290	0.623	0.277	0.735	0.741	0.832	0.209	0.211	0.217	0.622	0.635
PCB 49	25.34	25.45	25.29	0.533	0.984	0.343	0.915	0.916	0.712	0.950	0.950	0.472	0.880	0.881
PCB 52	29.90	29.99	29.87	0.088	0.992	0.143	0.947	0.950	0.351	0.926	0.927	0.107	0.924	0.928
PCB 37	60.87	64.08	64.76	0.102	0.373	0.081	0.342	0.392	0.070	0.063	0.115	0.313	0.848	0.851
PCB 70	34.80	37.18	37.48	0.364	0.202	0.233	0.191	0.236	0.292	0.053	0.072	0.520	0.871	0.872
**PCB 74**	34.76	37.28	37.80	0.214	0.128	0.167	0.167	0.225	**0.119**	**0.014**	**0.030**	0.362	0.763	0.769
PCB 87	39.47	40.61	40.42	0.055	0.728	0.078	0.550	0.573	0.801	0.262	0.262	0.088	0.921	0.924
**PCB 99**	32.33	33.20	33.84	0.249	0.339	0.249	0.472	0.494	**0.915**	**0.047**	**0.048**	0.226	0.583	0.601
PCB 114	29.70	31.42	31.29	0.504	0.357	0.340	0.241	0.273	0.218	0.169	0.187	0.735	0.933	0.933
PCB 101	31.01	32.43	32.07	0.558	0.651	0.692	0.450	0.452	0.340	0.528	0.535	0.482	0.805	0.806
PCB 81	39.00	41.32	41.41	0.382	0.585	0.255	0.444	0.458	0.942	0.277	0.278	0.323	0.977	0.977
PCB 77	39.12	42.12	43.26	0.729	0.171	0.504	0.214	0.226	0.814	0.067	0.068	0.631	0.622	0.624
PCB 151	27.34	28.32	28.18	0.397	0.483	0.330	0.270	0.304	0.098	0.305	0.335	0.915	0.893	0.893
PCB 119	31.05	32.63	33.67	0.571	0.215	0.354	0.290	0.308	0.373	0.084	0.095	0.800	0.523	0.524
PCB 105	31.97	34.18	34.45	0.569	0.147	0.410	0.122	0.144	0.242	0.075	0.090	0.978	0.846	0.846
PCB 123	31.39	33.13	33.88	0.480	0.367	0.335	0.328	0.347	0.215	0.168	0.187	0.983	0.713	0.713
PCB 153/157	29.29	30.65	30.48	0.583	0.595	0.718	0.327	0.331	0.293	0.443	0.452	0.560	0.916	0.916
PCB 118	34.04	35.67	36.21	0.231	0.427	0.177	0.349	0.392	0.059	0.166	0.218	0.892	0.794	0.794
PCB-SS	61.83	66.19	65.80	0.647	0.222	0.450	0.165	0.172	0.812	0.136	0.136	0.509	0.887	0.888
PCB 128/158	30.96	32.73	33.47	0.969	0.225	0.818	0.250	0.251	0.887	0.118	0.118	0.918	0.614	0.614
PCB 126	39.11	43.58	46.34	0.972	0.099	0.839	0.182	0.183	0.991	0.054	0.054	0.854	0.387	0.387
PCB 180	28.29	29.12	30.00	0.291	0.441	0.421	0.601	0.605	0.140	0.257	0.295	0.468	0.391	0.406

PCB 177	27.97	30.21	29.65	0.543	0.140	0.443	0.068	0.075	0.310	0.156	0.168	0.785	0.637	0.637
**PCB 138**	35.53	37.22	39.30	0.234	0.060	0.139	0.267	0.314	**0.280**	**0.010**	**0.025**	0.421	0.234	0.249
PCB 167	34.97	38.26	39.30	0.970	0.089	0.860	0.123	0.123	0.968	0.059	0.059	0.804	0.560	0.560
**PCB 187**	24.20	26.61	27.09	0.121	0.042	**0.193**	**0.009**	**0.029**	**0.089**	**0.048**	**0.102**	0.269	0.691	0.699
PCB 156	37.82	40.89	41.86	0.877	0.120	0.699	0.130	0.135	0.620	0.069	0.075	0.937	0.626	0.626
**PCB 168**	35.79	38.87	40.49	0.932	0.055	0.892	0.091	0.092	**0.829**	**0.044**	**0.046**	0.747	0.361	0.365
**PCB 183**	38.31	39.58	40.86	0.431	0.154	0.297	0.353	0.370	**0.685**	**0.041**	**0.042**	0.404	0.363	0.375
PCB 169	52.31	56.20	58.15	0.460	0.201	0.618	0.311	0.319	0.379	0.057	0.070	0.284	0.549	0.566
PCB 170	37.74	37.16	38.01	0.253	0.825	0.657	0.744	0.744	0.084	0.844	0.850	0.260	0.495	0.518
PCB 199	29.77	30.02	30.76	0.138	0.668	0.364	0.693	0.698	0.223	0.496	0.515	0.116	0.588	0.611
**PCB 189**	35.28	38.09	39.85	0.926	0.025	0.747	0.078	0.082	**0.980**	**0.024**	**0.024**	0.710	0.198	0.201
PCB 194	32.64	34.58	35.58	0.342	0.107	0.717	0.061	0.068	0.356	0.096	0.120	0.238	0.478	0.501

Table S7 ANOVA and IST analysis of recoveries of extracted target chemicals by EA/CH (1:1), DCM/HEX (4:1), and HEX/ACE (1:1) in spiked fish meat

Analytes	Extract solvent (%)			TOHOV	ANOVA	IST (ES1 vs. ES2)			IST (ES1 vs. ES3)			IST (ES2 vs. ES3)		
	ES1	ES2	ES3	*p*	*p*	p_L	p_e	p_i	p_L	p_e	p_i	p_L	p_e	p_i
**PAHs**														
Nap	95.95	108.73	96.81	0.092	0.252	0.076	0.162	0.138	0.066	0.917	0.897	0.781	0.211	0.211
PAH-SS1	129.16	116.52	115.51	0.357	0.373	0.511	0.166	0.133	0.234	0.283	0.221	0.391	0.919	0.920
Any	84.61	87.72	88.30	0.171	0.535	0.912	0.150	0.176	0.229	0.420	0.347	0.147	0.861	0.866
**Ace**	107.33	111.01	106.99	0.062	0.162	0.190	0.258	0.404	0.003	0.889	0.926	**0.340**	**0.036**	**0.063**
Fle	127.97	136.04	125.88	0.177	0.723	0.187	0.663	0.605	0.621	0.824	0.816	0.177	0.504	0.519
Phe	111.29	160.62	110.66	0.028	0.544	0.086	0.516	0.442	0.960	0.969	0.971	0.033	0.395	0.436
Ant	111.02	114.05	109.30	0.048	0.700	0.138	0.724	0.669	0.308	0.435	0.537	0.045	0.474	0.509
Flu	134.82	110.63	120.64	0.039	0.573	0.174	0.446	0.378	0.049	0.221	0.407	0.033	0.654	0.675
Pyr	65.33	59.30	75.17	0.661	0.332	0.446	0.632	0.607	0.672	0.377	0.384	0.602	0.186	0.190
PAH-SS2	92.46	82.67	83.21	0.485	0.430	0.417	0.329	0.292	0.942	0.223	0.262	0.367	0.942	0.943
Baa	85.73	85.52	85.43	0.097	0.987	0.057	0.797	0.848	0.249	0.908	0.890	0.074	0.965	0.966
Chr	93.79	94.13	95.96	0.125	0.779	0.757	0.867	0.859	0.149	0.635	0.570	0.124	0.614	0.628
BbF	138.42	139.71	133.61	0.071	0.476	0.999	0.863	0.869	0.004	0.348	0.541	0.070	0.234	0.284
BkF	133.49	131.07	126.66	0.029	0.403	0.791	0.721	0.727	0.001	0.155	0.383	0.030	0.330	0.379
BaP	108.88	105.42	103.61	0.017	0.028	0.046	0.119	0.281	0.010	0.038	0.194	0.378	0.087	0.095
IcdP	92.82	93.04	91.38	0.500	0.712	0.246	0.925	0.938	0.949	0.623	0.646	0.382	0.442	0.448
DiahA	85.74	87.15	85.17	0.985	0.832	0.872	0.722	0.730	0.883	0.888	0.889	0.982	0.580	0.580
BghiP	89.46	91.50	90.35	0.465	0.846	0.398	0.517	0.484	0.326	0.830	0.802	0.522	0.753	0.755
**PAHS**														
**OCP-SS**	82.52	75.57	94.99	0.418	0.013	0.740	0.253	0.254	**0.366**	**0.032**	**0.050**	**0.170**	**0.010**	**0.024**
α-HCH	89.12	101.59	94.27	0.053	0.151	0.063	0.122	0.182	0.245	0.089	0.111	0.117	0.330	0.365
HCB	90.53	90.87	89.39	0.591	0.476	0.808	0.749	0.750	0.506	0.431	0.435	0.384	0.300	0.310
β-HCH	83.05	89.00	88.88	0.038	0.440	0.056	0.371	0.418	0.089	0.389	0.427	0.214	0.940	0.941

γ-HCH	89.62	93.52	93.13	0.071	0.286	0.095	0.243	0.284	0.097	0.286	0.324	0.847	0.771	0.771
δ-HCH	88.55	89.76	91.57	0.114	0.836	0.146	0.847	0.852	0.115	0.628	0.645	0.887	0.542	0.543
HC	107.47	109.01	109.37	0.920	0.663	0.795	0.496	0.496	0.891	0.445	0.445	0.713	0.873	0.873
Ald	95.98	95.01	92.55	0.490	0.388	0.679	0.743	0.743	0.457	0.156	0.181	0.225	0.348	0.375
ID	93.79	81.63	90.45	0.009	0.458	0.033	0.349	0.395	0.316	0.215	0.239	0.022	0.480	0.517
cHCP	86.44	102.03	103.43	0.107	0.344	0.251	0.123	0.150	0.062	0.246	0.291	0.269	0.925	0.926
oCd	95.66	98.68	99.86	0.050	0.769	0.573	0.369	0.378	0.097	0.580	0.596	0.049	0.868	0.875
tHCP	91.69	96.98	96.41	0.261	0.467	0.361	0.194	0.204	0.162	0.363	0.386	0.404	0.916	0.917
tCd	92.49	97.81	87.54	0.020	0.375	0.828	0.084	0.084	0.044	0.574	0.599	0.042	0.273	0.323
o,p’-DDE	105.77	106.49	70.16	0.006	0.409	0.765	0.844	0.844	0.021	0.369	0.417	0.020	0.360	0.409
ES I	101.09	103.74	100.83	0.220	0.475	0.217	0.402	0.422	0.183	0.930	0.932	0.986	0.147	0.148
cCd	92.99	93.69	91.64	0.196	0.722	0.459	0.827	0.829	0.107	0.631	0.650	0.217	0.311	0.344
Dld	133.53	133.07	131.11	0.341	0.852	0.542	0.934	0.934	0.206	0.628	0.642	0.289	0.597	0.604
p,p’-DDE	93.28	93.75	88.06	0.457	0.194	0.190	0.882	0.885	0.608	0.207	0.210	0.513	0.089	0.106
o,p’-DDD	92.31	92.58	90.17	0.210	0.660	0.364	0.941	0.943	0.158	0.530	0.560	0.155	0.185	0.223
Ed	127.98	126.17	125.52	0.240	0.785	0.244	0.691	0.704	0.227	0.588	0.608	0.966	0.740	0.740
**ES II**	100.07	73.63	103.12	0.010	0.000	**0.031**	**0.005**	**0.025**	0.026	0.541	0.571	**0.696**	**0.000**	**0.000**
p,p’-DDD	116.16	113.01	112.38	0.814	0.693	0.662	0.558	0.563	0.632	0.486	0.492	0.916	0.877	0.877
**o,p’-DDT**	151.72	155.11	142.09	0.110	0.159	0.081	0.626	0.641	0.225	0.241	0.257	**0.287**	**0.035**	**0.044**
p,p’-DDT	158.48	159.91	155.66	0.047	0.575	0.193	0.778	0.782	0.038	0.546	0.574	0.097	0.139	0.185
MOC	112.73	125.82	117.81	0.232	0.959	0.424	0.824	0.827	0.136	0.922	0.926	0.268	0.796	0.804
Mirex	102.04	102.28	104.99	0.083	0.838	0.269	0.972	0.973	0.038	0.632	0.653	0.219	0.495	0.518
**PBDEs**														
PBDE 17	101.86	105.63	105.52	0.662	0.184	0.490	0.160	0.180	0.863	0.077	0.077	0.546	0.965	0.966
PBDE 28	99.55	102.37	102.62	0.243	0.287	0.653	0.285	0.287	0.241	0.129	0.161	0.104	0.899	0.903
**PBDE-SS**	101.58	105.13	103.35	0.088	0.060	**0.053**	**0.044**	**0.087**	0.186	0.089	0.120	0.272	0.267	0.281
PBDE 71	97.96	98.82	99.80	0.159	0.552	0.828	0.684	0.684	0.082	0.279	0.333	0.100	0.498	0.531
PBDE 47	95.39	96.92	98.44	0.581	0.321	0.562	0.498	0.504	0.396	0.193	0.217	0.621	0.349	0.352
PBDE 66	96.24	96.07	98.21	0.517	0.676	0.633	0.956	0.957	0.323	0.504	0.519	0.430	0.357	0.365
PBDE 100	96.06	95.79	98.02	0.645	0.541	0.681	0.912	0.913	0.410	0.413	0.428	0.575	0.261	0.268

PBDE 99	95.71	94.15	97.92	0.396	0.588	0.326	0.710	0.716	0.318	0.602	0.611	0.928	0.215	0.215
PBDE 85	96.68	94.31	99.77	0.235	0.524	0.147	0.660	0.669	0.224	0.580	0.588	0.975	0.176	0.178
PBDE 154	95.24	93.00	97.14	0.517	0.524	0.218	0.554	0.562	0.526	0.650	0.651	0.718	0.234	0.241
PBDE 153	93.01	90.31	95.50	0.249	0.587	0.119	0.615	0.629	0.415	0.679	0.682	0.401	0.228	0.247
PBDE 138	85.94	81.96	87.16	0.094	0.597	0.050	0.467	0.504	0.602	0.854	0.855	0.077	0.254	0.309
PBDE 183	85.35	81.01	84.10	0.131	0.722	0.089	0.456	0.496	0.700	0.859	0.860	0.056	0.481	0.518
PBDE 190	78.74	72.05	75.84	0.257	0.586	0.157	0.354	0.391	0.530	0.714	0.717	0.204	0.447	0.467
PBDE 209	70.49	60.55	72.69	0.096	0.673	0.034	0.473	0.507	0.821	0.904	0.904	0.092	0.369	0.411
**PCBs**														
PCB 18	76.08	77.65	78.34	0.415	0.728	0.510	0.651	0.654	0.462	0.368	0.373	0.260	0.827	0.831
PCB 44	75.17	76.49	78.22	0.290	0.654	0.277	0.735	0.741	0.832	0.230	0.232	0.217	0.654	0.665
PCB 49	74.87	74.81	74.86	0.533	1.000	0.343	0.986	0.987	0.712	0.998	0.998	0.472	0.988	0.988
PCB 52	90.23	90.29	89.82	0.088	0.986	0.143	0.988	0.989	0.351	0.729	0.732	0.107	0.903	0.908
PCB 37	92.36	97.03	98.14	0.102	0.390	0.081	0.360	0.408	0.070	0.067	0.119	0.313	0.837	0.840
PCB 70	90.70	96.91	97.56	0.364	0.207	0.233	0.191	0.236	0.292	0.056	0.075	0.520	0.889	0.890
**PCB 74**	87.70	93.80	95.26	0.214	0.136	0.167	0.181	0.238	**0.119**	**0.015**	**0.031**	0.362	0.738	0.745
PCB 87	92.30	94.85	94.16	0.055	0.763	0.078	0.568	0.590	0.801	0.339	0.339	0.088	0.876	0.881
PCB 99	89.86	92.15	93.73	0.249	0.387	0.249	0.491	0.512	0.915	0.059	0.060	0.226	0.626	0.642
PCB 114	86.91	91.92	91.55	0.504	0.357	0.340	0.241	0.273	0.218	0.169	0.187	0.735	0.933	0.933
PCB 101	95.16	99.31	98.26	0.558	0.676	0.692	0.471	0.473	0.340	0.547	0.554	0.482	0.814	0.815
PCB 81	100.32	106.29	106.52	0.382	0.585	0.255	0.444	0.458	0.942	0.277	0.278	0.323	0.977	0.977
PCB 77	92.85	99.82	102.51	0.729	0.179	0.504	0.222	0.235	0.814	0.070	0.071	0.631	0.621	0.624
PCB 151	90.81	94.08	93.61	0.397	0.483	0.330	0.270	0.304	0.098	0.305	0.335	0.915	0.893	0.893
PCB 119	85.19	89.53	92.38	0.571	0.215	0.354	0.290	0.308	0.373	0.084	0.095	0.800	0.523	0.524
PCB 105	87.73	93.51	94.21	0.569	0.168	0.410	0.135	0.157	0.242	0.085	0.100	0.978	0.858	0.858
PCB 123	90.11	95.12	97.27	0.480	0.367	0.335	0.328	0.347	0.215	0.168	0.187	0.983	0.713	0.713
PCB 153/157	92.69	96.87	96.12	0.583	0.624	0.718	0.340	0.344	0.293	0.482	0.490	0.560	0.885	0.885
PCB 118	98.79	103.51	105.09	0.231	0.427	0.177	0.349	0.392	0.059	0.166	0.218	0.892	0.794	0.794
PCB-SS	92.16	98.67	98.08	0.647	0.222	0.450	0.165	0.172	0.812	0.136	0.136	0.509	0.887	0.888
PCB 128/158	91.23	96.51	98.42	0.969	0.236	0.818	0.245	0.247	0.887	0.126	0.126	0.918	0.658	0.659

PCB 126	86.35	96.22	102.31	0.972	0.099	0.839	0.182	0.183	0.991	0.054	0.054	0.854	0.387	0.387
PCB 180	94.42	97.19	100.12	0.291	0.441	0.421	0.601	0.605	0.140	0.257	0.295	0.468	0.391	0.406
PCB 177	91.34	98.64	96.82	0.543	0.140	0.443	0.068	0.075	0.310	0.156	0.168	0.785	0.637	0.637
**PCB 138**	93.22	97.66	103.11	0.234	0.060	0.139	0.267	0.314	**0.280**	**0.010**	**0.025**	0.421	0.234	0.249
PCB 167	88.78	97.13	99.76	0.970	0.089	0.860	0.123	0.123	0.968	0.059	0.059	0.804	0.560	0.560
PCB 187	89.52	98.43	100.21	0.121	0.042	0.193	0.009	0.029	0.089	0.048	0.102	0.269	0.691	0.699
PCB 156	91.51	98.94	101.28	0.877	0.120	0.699	0.130	0.135	0.620	0.069	0.075	0.937	0.626	0.626
**PCB 168**	90.73	98.53	102.63	0.932	0.055	0.892	0.091	0.092	**0.829**	**0.044**	**0.046**	0.747	0.361	0.365
**PCB 183**	100.23	103.54	106.89	0.431	0.154	0.297	0.353	0.370	**0.685**	**0.041**	**0.042**	0.404	0.363	0.375
PCB 169	116.61	125.27	129.63	0.460	0.201	0.618	0.311	0.319	0.379	0.057	0.070	0.284	0.549	0.566
PCB 170	124.08	122.19	124.98	0.253	0.825	0.657	0.744	0.744	0.084	0.844	0.850	0.260	0.495	0.518
PCB 199	104.15	105.04	107.63	0.138	0.668	0.364	0.693	0.698	0.223	0.496	0.515	0.116	0.588	0.611
**PCB 189**	104.72	113.08	118.30	0.926	0.025	0.747	0.078	0.082	**0.980**	**0.024**	**0.024**	0.710	0.198	0.201
PCB 194	104.69	110.90	114.12	0.342	0.107	0.717	0.061	0.068	0.356	0.096	0.120	0.238	0.478	0.501

Table S17 Recoveries (RV), relative standard deviation (RSD), quality judgment (QJ) of target chemicals in spiked fish meat extracted by EA/CH (1:1), DCM/HEX (4:1), and HEX/ACE(1:1)

Analytes	EA/CH (1:1)			DCM/HEX (4:1)			HEX/ACE(1:1)		
	RV, %	RSD, %	QJ	RV, %	RSD, %	QJ	RV, %	RSD, %	QJ
**PAHs**									
Nap	95.9	0.8	G	110.1	8.4	G	96.7	10.6	G
PAH-SS1	125.1	4.3	G	123.3	3.0	G	119.4	9.2	G
Any	84.6	2.1	G	87.1	2.0	G	88.5	5.8	G
Ace	107.3	3.8	G	111.2	1.9	G	107.4	0.8	G
Fle	128.0	6.3	G	136.3	16.0	B	128.0	7.9	G
Phe	111.3	15.2	G	166.7	53.6	B	127.5	12.6	G
Ant	111.0	2.5	G	114.9	9.0	G	110.4	1.5	G
Flu	134.8	11.5	B	124.1	28.6	G	128.5	4.4	G
Pyr	65.3	15.1	B	71.3	19.0	G	79.9	13.4	G
PAH-SS2	121.0	23.1	G	109.4	17.7	G	102.1	17.3	G
Baa	85.7	1.4	G	86.1	0.6	G	85.7	3.6	G
Chr	93.8	1.8	G	95.7	2.2	G	96.5	5.6	G
BbF	138.4	5.6	B	140.0	5.2	B	133.7	1.4	B
BkF	133.5	5.0	B	131.6	5.2	B	126.7	0.8	G
BaP	108.9	2.4	G	108.4	1.1	G	104.3	0.8	G
IcdP	92.8	3.3	G	93.1	2.1	G	91.5	3.1	G
DiahA	85.7	4.6	G	87.2	4.5	G	85.2	4.9	G
BghiP	89.5	2.6	G	91.6	3.7	G	90.4	5.4	G
**OCPs**									
OCP-SS	88.4	9.4	G	81.6	9.5	G	94.2	10.3	G
α-HCH	89.1	2.1	G	101.6	10.7	G	94.3	3.8	G
HCB	90.5	1.5	G	90.9	1.3	G	89.4	2.0	G
β-HCH	83.0	12.3	G	89.0	1.2	G	88.9	2.7	G
γ-HCH	89.6	5.2	G	93.5	1.6	G	93.1	1.7	G
δ-HCH	88.5	10.8	G	89.8	4.0	G	91.6	3.3	G
HC	107.5	2.5	G	109.0	2.2	G	109.4	2.6	G
Ald	96.0	3.2	G	95.0	3.9	G	92.5	1.7	G
ID	93.8	3.7	G	81.6	24.0	G	90.4	2.0	G
cHCP	86.4	6.9	G	102.0	12.2	G	103.4	20.1	G
oCd	95.7	4.5	G	98.7	2.8	G	99.9	11.3	G
tHCP	91.7	3.6	G	97.0	5.0	G	96.4	7.5	G
tCd	92.5	3.2	G	97.8	2.8	G	87.5	15.7	G
o,p’-DDE	105.8	4.5	G	106.5	3.4	G	105.2	2.9	G
ES I	101.1	4.4	G	103.7	2.0	G	100.8	1.9	G
cCd	93.0	4.7	G	93.7	3.1	G	91.6	1.2	G
Dld	133.5	5.6	B	133.1	3.9	B	131.1	2.2	B
p,p’-DDE	93.3	5.0	G	93.8	2.4	G	88.1	4.3	G
o,p’-DDD	92.3	5.8	G	92.6	2.6	G	90.2	1.0	G

Ed	128.0	5.4	G	126.2	1.9	G	125.5	1.7	G
ES II	100.1	7.8	G	73.6	1.8	G	103.1	1.0	G
p,p’-DDD	116.2	6.2	G	113.0	4.1	G	112.4	4.1	G
o,p’-DDT	151.7	6.9	B	155.1	2.5	B	142.1	4.3	B
p,p’-DDT	158.5	4.6	B	159.9	2.4	B	155.7	0.8	B
MOC	612.7	13.5	B	625.8	7.6	B	617.8	2.5	B
Mirex	102.0	9.5	G	102.3	5.8	G	105.0	2.0	G
**PBDEs**									
PBDE 17	101.9	1.8	G	105.6	3.1	G	105.5	1.9	G
PBDE 28	99.5	2.6	G	102.4	3.0	G	102.6	1.1	G
PBDE-SS	102.1	1.6	G	101.6	3.6	G	100.8	2.6	G
PBDE 71	98.0	2.6	G	98.8	2.3	G	99.8	0.4	G
PBDE 47	95.4	3.2	G	96.9	2.0	G	98.4	1.6	G
PBDE 66	96.2	4.4	G	96.1	3.1	G	98.2	2.0	G
PBDE 100	96.1	3.4	G	95.8	2.5	G	98.0	1.8	G
PBDE 99	95.7	6.3	G	94.1	3.3	G	97.9	3.2	G
PBDE 85	96.7	8.0	G	94.3	4.0	G	99.8	4.4	G
PBDE 154	95.2	5.5	G	93.0	3.2	G	97.1	4.3	G
PBDE 153	93.0	8.6	G	90.3	3.4	G	95.5	5.8	G
PBDE 138	85.9	9.9	G	82.0	1.4	G	87.2	7.7	G
PBDE 183	85.3	10.7	G	81.0	0.6	G	84.1	8.2	G
PBDE 190	78.7	13.5	G	72.1	4.4	G	75.8	9.4	G
PBDE 209	70.5	30.3	B	60.6	6.7	B	72.7	28.0	G
**PCBs**									
PCB 18	76.1	4.1	G	77.7	5.9	G	78.3	2.9	G
PCB 44	75.2	3.8	G	76.5	7.4	G	78.2	3.1	G
PCB 49	74.9	3.6	G	74.8	5.8	G	74.9	4.1	G
PCB 52	90.2	1.8	G	90.3	6.9	G	89.8	1.2	G
PCB 37	92.4	0.9	G	97.0	8.0	G	98.1	4.0	G
PCB 70	90.7	2.3	G	96.9	6.7	G	97.6	4.0	G
PCB 74	87.7	1.5	G	93.8	6.8	G	95.3	3.1	G
PCB 87	92.3	2.2	G	94.8	7.2	G	94.2	2.3	G
PCB 99	89.9	2.1	G	92.2	5.3	G	93.7	1.8	G
PCB 114	86.9	2.8	G	91.9	6.4	G	91.6	4.5	G
PCB 101	95.2	7.3	G	99.3	5.8	G	98.3	4.4	G
PCB 81	100.3	5.6	G	106.3	10.2	G	106.5	6.0	G
PCB 77	92.8	4.8	G	99.8	7.1	G	102.5	5.0	G
PCB 151	90.8	1.7	G	94.1	4.4	G	93.6	4.1	G
PCB 119	85.2	3.5	G	89.5	6.0	G	92.4	4.9	G
PCB 105	87.7	2.8	G	93.5	5.1	G	94.2	4.5	G
PCB 123	90.1	4.0	G	95.1	7.3	G	97.3	6.6	G
PCB 153/157	92.7	4.3	G	96.9	5.5	G	96.1	6.8	G
PCB 118	98.8	1.6	G	103.5	7.3	G	105.1	5.9	G
PCB-SS	79.6	4.8	G	93.2	5.8	G	97.2	4.1	G
PCB 128/158	91.2	4.8	G	96.5	5.3	G	98.4	4.8	G
PCB 126	86.3	8.1	G	96.2	8.3	G	102.3	7.3	G

PCB 180	94.4	7.5	G	97.2	4.8	G	100.1	2.5	G
PCB 177	91.3	3.2	G	98.6	4.2	G	96.8	4.7	G
PCB 138	93.2	1.6	G	97.7	5.9	G	103.1	3.3	G
PCB 167	88.8	6.0	G	97.1	5.3	G	99.8	5.0	G
PCB 187	89.5	1.1	G	98.4	3.1	G	100.2	6.5	G
PCB 156	91.5	4.5	G	98.9	5.4	G	101.3	5.5	G
PCB 168	90.7	4.9	G	98.5	4.2	G	102.6	5.4	G
PCB 183	100.2	2.5	G	103.5	4.7	G	106.9	2.7	G
PCB 169	116.6	6.3	G	125.3	8.5	G	129.6	3.3	G
PCB 170	124.1	5.7	G	122.2	5.0	G	125.0	1.7	G
PCB 199	104.2	3.0	G	105.0	1.7	G	107.6	6.9	G
PCB 189	104.7	4.6	G	113.1	3.3	G	118.3	3.8	G
PCB 194	104.7	3.2	G	110.9	2.2	G	114.1	5.9	G

Note: G – good, B – bad

Table S9 GC/MS or GC/MS/MS abundance of target chemicals at every fraction of GPC from the 10th min

Fraction No.	1	2	3	4	5	6	7	8	9	10
Time, min	10~13	13~16	16~19	19~22	22~25	25~28	28~31	31~34	34~37	37~40
**PAHs**										
PAH-IS1		4622	11077							
Nap	638	3122	7954	515	584	435			513	
PAH-IS2	2323	517	8742	85						
PAH-SS1										
Any		664	12254							
Ace		425	6444	179	107	70	84		103	
Fle	172	899	8499	194	134	154	133	105	196	
PAH-IS3		141	5785	883						
Phe	542	690	9943	2660	476	510	443	328	712	337
Ant	82	213	8183	1728	41	30	26	21	66	15
Flu	579	157	2813	7150	127	110			105	
PAH-IS4			282	3602	241					
Pyr	801	275	1157	9014	760	206	263	142	259	189
PAH-SS2										
Ret		808	776							
BcP			744	3894	78					
CcdP			54	879	2429					
BaA	174		343	3231	113					
Chr	122		252	3740	427					
PAH-IS5				50	546	498				
BbF				896	1385					
BkF				1071	981					
BeP				112	1659	650				
BaP				108	1365	120				
Per					505	674				
IcdP					580	379				
DahA				329	788					
BghiP					86	403	609			
Antt					63	343	395			
DiBalP					63	280	361			
DiBaeF					213	396	91			
Cor						75	425	313		
DiBaeP						218	470			
DiBaiP						146	244			
DiBahP						140	346			
**OCPs**										
OCP-IS	15	191	3530	872						
OCP-SS		45	1040	103						

α-HCH	310	3110	83				
HCB	70	601	13997	8317	37		
β-HCH	869	131					
γ-HCH	172	2256	118				
δ-HCH	2184	525					
HC	33	458	33				
Ald	10	352	135				
ID	114	3987	3109				
cHCP		43					
oCd	30	66					
tHCP		79					
tCd	645	851					
o,p’-DDE		132	48				
ES I	45	795	138				
cCd	132	433	51				
Dld	48	1682	2003	22			
p,p’-DDE	143	4065	1745	12			
o,p’-DDD	307	1325	67				
Ed	54	896	3198	90			
ES II	131	1042	62				
p,p’-DDD	852	1634	34				
o,p’-DDT	852	1730	622				
p,p’-DDT	124	1471	119				
MOC	6	335	532	6	19		
Mirex	8	350	348				
**PBDEs**							
PBDE-IS	1219	33691	14939	181	17		
PBDE 17		127	1702	627			
PBDE 28		129	1514	483			
PBDE-SS	54	1894	2198	36			
PBDE 71		169	1573	458			
PBDE 47		109	1301	584			
PBDE 66		71	1104	547			
PBDE 100		144	1581	741			
PBDE 99		129	1444	575			
PBDE 85			543	1180			
PBDE 154		258	1745	394			
PBDE 153		259	1560	283			
PBDE 138		37	596	1018	30		
PBDE 183		280	1226	467			
PBDE 190		21	328	1095	150		
PBDE 209			21	484	3475	2155	197
**PCBs**							
PCB 18	218	4230	7760	79			
PCB 44	42	817	727				
PCB 49	44	847	551				

PCB 52	18	631	967	
PCB 37		95	1482	25
PCB 70		527	1724	44
PCB 74		711	1737	58
PCB 99	68	1262	1040	
PCB 87	71	1725	1009	
PCB 114	109	2159	1106	
PCB 101	62	1952	2336	
PCB 81		86	775	94
PCB 77		167	1462	
PCB 151	103	1792	501	
PCB 119		·	1295	
PCB 105	38	620	1513	30
PCB 123		405	1666	110
PCB 153/157	86	1482	823	
PCB 118		237	718	43
PCB-SS		437	457	
PCB 128/158	118	2373	2103	32
PCB 126		127	544	
PCB 180	47	682	197	
PCB 177		632	217	
PCB 138		282	436	
PCB 167		326	583	
PCB 187	66	996	488	
PCB 156		206	678	53
PCB 168		174	576	58
PCB 183		630	648	
PCB 169		114	118	
PCB 170		166	226	
PCB 199	35	459	156	
PCB 189		533	1414	56
PCB-IS		971	441	
PCB 194	35	629	739	37

Table S10 ANOVA and IST analysis of recoveries of PAHs in spiked fish meat cleaned up by SPE cartridge (SC) and chromatography column (CC)

Analytes	SC, %	CC, %	TOHOV	ANOVA	IST (CS vs. CC)			ANOVA	IST	QJ
			p	*p*	p_L	p_e	p_i			
**PAHs**										
Nap	108.1	97.1	0.102	0.201	0.102	0.201	0.235	U	U	U
PAH-SS1	89.1	87.5	0.112	0.802	0.112	0.802	0.813	U	U	U
Any	88.1	105.4	0.053	0.299	0.053	0.299	0.355	U	U	U
Ace	93.5	92.0	0.034	0.703	0.034	0.703	0.719	0	U	U
Fle	140.3	100.5	0.130	0.001	0.130	0.001	0.016	S	S	CC
Phe	152.4	107.2	0.060	0.071	0.060	0.071	0.119	U	U	U
Ant	103.4	94.1	0.434	0.015	0.434	0.015	0.019	S	S	SC
Flu	138.6	115.1	0.374	0.255	0.374	0.255	0.284	U	U	U
Pyr	95.2	118.5	0.023	0.170	0.023	0.170	0.235	0	U	U
PAH-SS2	84.7	99.0	0.147	0.016	0.147	0.016	0.053	S	S	CC
Ret	83.8	111.0	0.350	0.002	0.350	0.002	0.003	S	S	CC
BcP	91.7	108.1	0.104	0.001	0.104	0.001	0.006	S	S	CC
CcdP	87.1	124.9	0.239	0.000	0.239	0.000	0.004	S	S	SC
BaA	97.7	98.1	0.989	0.755	0.989	0.755	0.755	U	U	U
Chr	95.3	100.6	0.672	0.011	0.672	0.011	0.012	S	S	CC
BbF	98.6	99.3	0.165	0.822	0.165	0.822	0.826	U	U	U
BkF	108.8	94.3	0.055	0.003	0.055	0.003	0.019	S	S	CC
BeP	103.4	102.9	0.090	0.856	0.090	0.856	0.861	U	U	U
BaP	96.2	95.5	0.072	0.741	0.072	0.741	0.753	U	U	U
Per	92.6	92.9	0.197	0.870	0.197	0.870	0.873	U	U	U
IcdP	110.9	84.9	0.396	0.001	0.396	0.001	0.002	S	S	SC
DahA	109.4	82.6	0.061	0.004	0.061	0.004	0.024	S	S	SC
BghiP	115.4	92.2	0.061	0.012	0.061	0.012	0.035	S	S	CC
Antt	98.3	73.0	0.061	0.012	0.061	0.012	0.038	S	S	SC
DiBalP	79.1	88.8	0.050	0.349	0.050	0.349	0.388	U	U	U
DiBaeF	82.3	79.1	0.028	0.793	0.028	0.793	0.805	0	U	U
Cor	93.9	83.8	0.019	0.465	0.019	0.465	0.504	0	U	U
DiBaeP	86.4	80.9	0.024	0.667	0.024	0.667	0.687	0	U	U
DiBaiP	68.7	70.7	0.048	0.855	0.048	0.855	0.862	0	U	U
DiBahP	62.2	62.2	0.027	0.999	0.027	0.999	0.999	0	U	U
**OCPs**										
OCP-SS	77.0	68.7	0.049	0.105	0.049	0.105	0.156	0	U	U
α-HCH	93.5	101.3	0.034	0.340	0.034	0.340	0.392	0	U	U
HCB	89.3	96.1	0.163	0.094	0.163	0.094	0.156	U	U	U
β-HCH	87.3	91.2	0.183	0.195	0.183	0.195	0.224	U	U	U
γ-HCH	92.9	99.8	0.132	0.124	0.132	0.124	0.186	U	U	U
δ-HCH	87.5	96.1	0.222	0.043	0.222	0.043	0.078	S	S	CC
HC	85.8	95.7	0.093	0.049	0.093	0.049	0.098	S	S	CC

Ald	86.5	97.5	0.202	0.028	0.202	0.028	0.052	S	S	CC
ID	88.4	97.8	0.367	0.008	0.367	0.008	0.015	S	S	CC
cHCP	89.7	87.9	0.042	0.720	0.042	0.720	0.734	0	U	U
oCd	91.1	94.8	0.234	0.167	0.234	0.167	0.186	U	U	U
tHCP	85.4	95.6	0.188	0.097	0.188	0.097	0.138	U	U	U
tCd	87.5	98.3	0.231	0.023	0.231	0.023	0.049	S	S	CC
o,p'-DDE	93.2	102.8	0.122	0.123	0.122	0.123	0.186	U	U	U
ES I	116.8	99.5	0.537	0.004	0.537	0.004	0.007	S	S	CC
cCd	83.3	95.1	0.298	0.017	0.298	0.017	0.036	S	S	CC
Dld	104.6	101.4	0.174	0.297	0.174	0.297	0.322	U	U	U
p,p'-DDE	81.8	94.3	0.250	0.053	0.250	0.053	0.088	U	U	U
o,p'-DDD	84.6	98.9	0.266	0.041	0.266	0.041	0.069	S	S	CC
Ed	92.2	95.8	0.705	0.203	0.705	0.203	0.212	U	U	U
ES II	85.2	94.9	0.870	0.039	0.870	0.039	0.041	S	S	CC
p,p'-DDD	84.3	97.7	0.278	0.216	0.278	0.216	0.250	U	U	U
o,p'-DDT	84.4	94.9	0.228	0.180	0.228	0.180	0.226	U	U	U
p,p'-DDT	108.1	98.6	0.078	0.866	0.078	0.866	0.873	U	U	U
MOC	51.3	95.6	0.594	0.001	0.594	0.001	0.002	S	S	CC
Mirex	66.1	98.6	0.555	0.019	0.555	0.019	0.022	S	S	CC
**PBDEs**										
PBDE 17	96.3	98.3	0.050	0.453	0.050	0.453	0.493	U	U	U
PBDE 28	97.0	96.5	0.840	0.900	0.840	0.900	0.900	U	U	U
PBDE-SS	93.3	96.3	0.052	0.020	0.052	0.020	0.051	S	S	CC
PBDE 71	95.3	93.4	0.905	0.296	0.905	0.296	0.296	U	U	U
PBDE 47	96.7	90.0	0.093	0.173	0.093	0.173	0.218	U	U	U
PBDE 66	99.4	89.4	0.146	0.005	0.146	0.005	0.012	S	S	SC
PBDE 100	97.9	95.0	0.661	0.165	0.661	0.165	0.174	U	U	U
PBDE 99	100.3	87.9	0.087	0.008	0.087	0.008	0.025	S	S	SC
PBDE 85	106.0	84.0	0.350	0.009	0.350	0.009	0.019	S	S	SC
PBDE 154	101.7	88.0	0.397	0.004	0.397	0.004	0.011	S	S	SC
PBDE 153	105.9	79.2	0.261	0.002	0.261	0.002	0.012	S	S	SC
PBDE 138	105.2	74.7	0.066	0.002	0.066	0.002	0.010	S	S	SC
PBDE 183	103.0	75.5	0.324	0.002	0.324	0.002	0.010	S	S	SC
PBDE 190	106.9	63.4	0.151	0.001	0.151	0.001	0.008	S	S	SC
PBDE 209	149.8	48.1	0.312	0.001	0.312	0.001	0.003	S	S	SC
**PCBs**										
PCB 18	74.1	100.3	0.282	0.006	0.282	0.006	0.019	S	S	CC
PCB 44	71.8	101.1	0.149	0.004	0.149	0.004	0.011	S	S	CC
PCB 49	70.1	106.2	0.121	0.002	0.121	0.002	0.011	S	S	CC
PCB 52	83.2	105.5	0.238	0.028	0.238	0.028	0.041	S	S	CC
PCB 37	86.0	81.3	0.135	0.791	0.135	0.791	0.803	U	U	U
PCB 70	86.6	99.2	0.063	0.059	0.063	0.059	0.099	U	U	U
PCB 74	82.4	97.6	0.119	0.109	0.119	0.109	0.151	U	U	U
PCB 87	83.1	107.2	0.461	0.012	0.461	0.012	0.016	S	S	CC
PCB 99	80.7	101.1	0.348	0.005	0.348	0.005	0.008	S	S	CC
PCB 114	82.2	103.5	0.323	0.014	0.323	0.014	0.021	S	S	CC

PCB 101	85.7	104.0	0.052	0.125	0.052	0.125	0.190	U	U	U
PCB 81	89.4	37.5	0.436	0.004	0.436	0.004	0.010	S	S	SC
PCB 77	84.9	72.2	0.155	0.422	0.155	0.422	0.453	U	U	U
PCB 151	85.6	100.5	0.194	0.003	0.194	0.003	0.011	S	S	CC
PCB 119	79.7	89.6	0.256	0.068	0.256	0.068	0.083	U	U	U
PCB 105	81.6	82.0	0.752	0.898	0.752	0.898	0.898	U	U	U
PCB 123	84.7	87.5	0.174	0.723	0.174	0.723	0.729	U	U	U
PCB 153/157	85.7	90.5	0.064	0.570	0.064	0.570	0.592	U	U	U
PCB 118	88.4	79.5	0.062	0.400	0.062	0.400	0.432	U	U	U
PCB-SS	97.4	85.5	0.124	0.120	0.124	0.120	0.186	U	U	U
PCB 128/158	93.8	92.1	0.668	0.631	0.668	0.631	0.632	U	U	U
PCB 126	80.0	75.1	0.353	0.304	0.353	0.304	0.313	U	U	U
PCB 180	88.9	86.0	0.140	0.794	0.140	0.794	0.806	U	U	U
PCB 177	84.8	90.2	0.121	0.026	0.121	0.026	0.046	S	S	CC
PCB 138	88.5	92.9	0.124	0.516	0.124	0.516	0.532	U	U	U
PCB 167	84.1	80.2	0.542	0.473	0.542	0.473	0.482	U	U	U
PCB 187	89.2	92.0	0.112	0.598	0.112	0.598	0.620	U	U	U
PCB 156	83.1	77.1	0.213	0.402	0.213	0.402	0.417	U	U	U
PCB 168	85.5	88.3	0.202	0.756	0.202	0.756	0.768	U	U	U
PCB 183	94.0	90.6	0.176	0.592	0.176	0.592	0.610	U	U	U
PCB 169	114.7	101.0	0.042	0.366	0.042	0.366	0.412	0	U	U
PCB 170	120.7	91.6	0.134	0.052	0.134	0.052	0.109	U	U	U
PCB 199	103.2	102.9	0.115	0.969	0.115	0.969	0.970	U	U	U
PCB 189	100.4	88.0	0.322	0.023	0.322	0.023	0.033	S	S	SC
PCB 194	96.4	109.4	0.201	0.327	0.201	0.327	0.368	U	U	U

Note: U – no significance, S – significance, 0 – unsuitable for ANOVA or IST, QJ – quality judgment

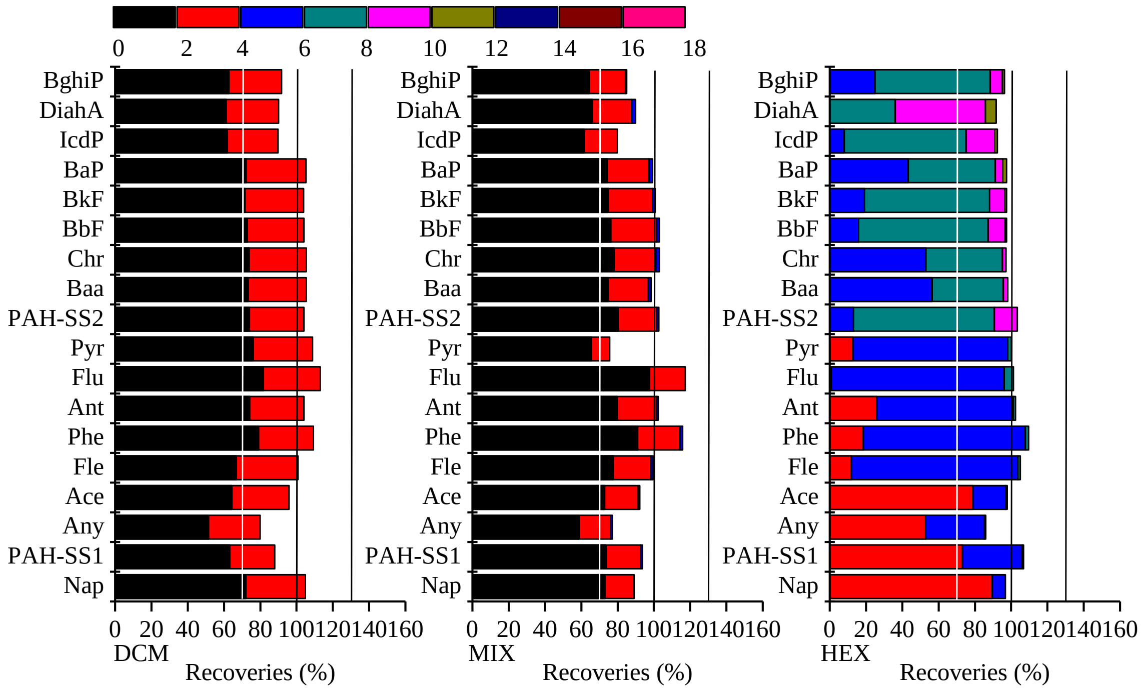

Fig. S1 Recoveries of PAHs at each SPE eluate (MIX – DCM/HEX (1:1, v/v))

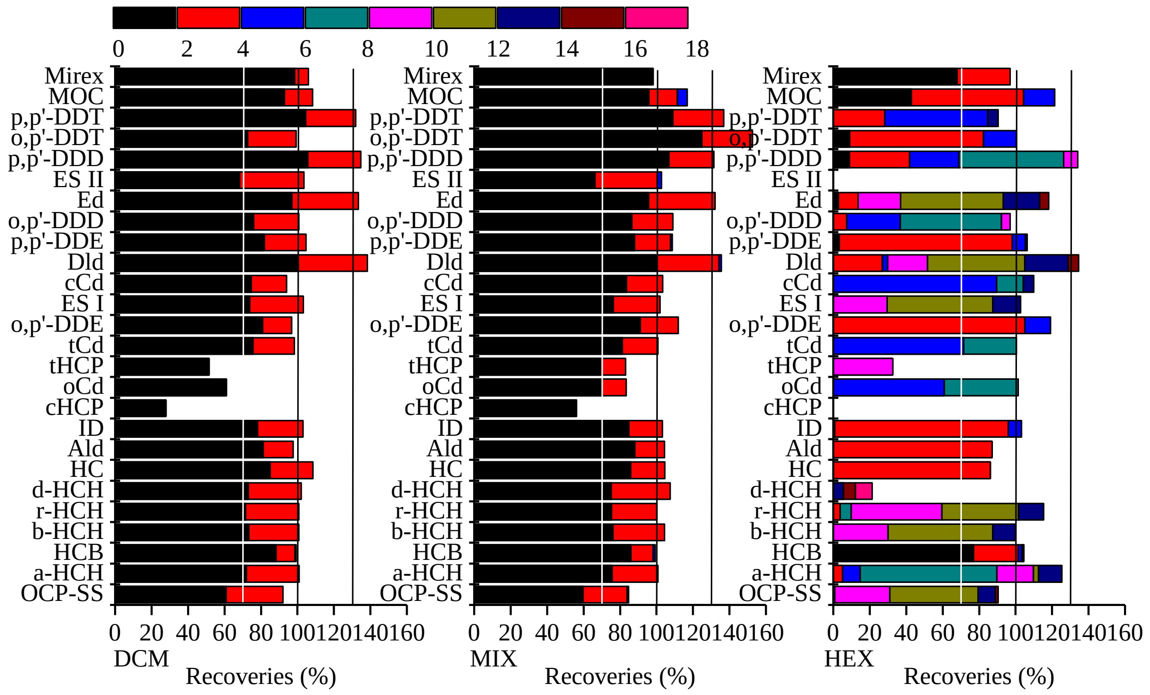

Fig. S2 Recoveries of OCPs at each SPE eluate (MIX – DCM/HEX (1:1, v/v))

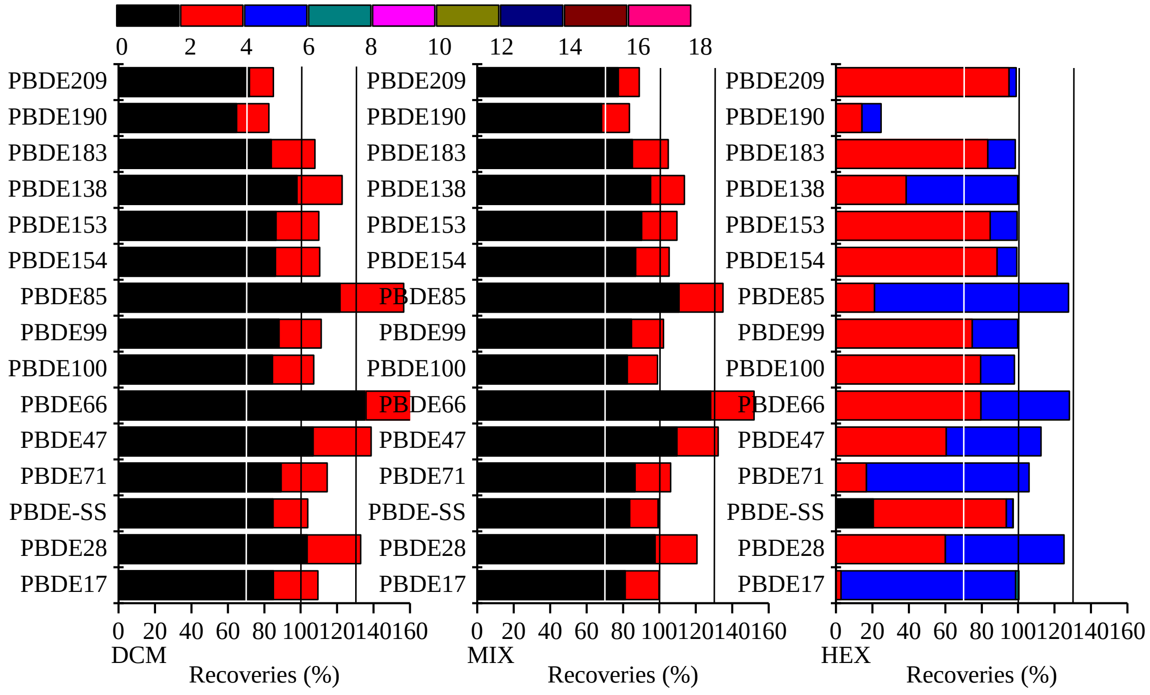

Fig. S3 Recoveries of PBDEs at each SPE eluate (MIX – DCM/HEX (1:1, v/v))

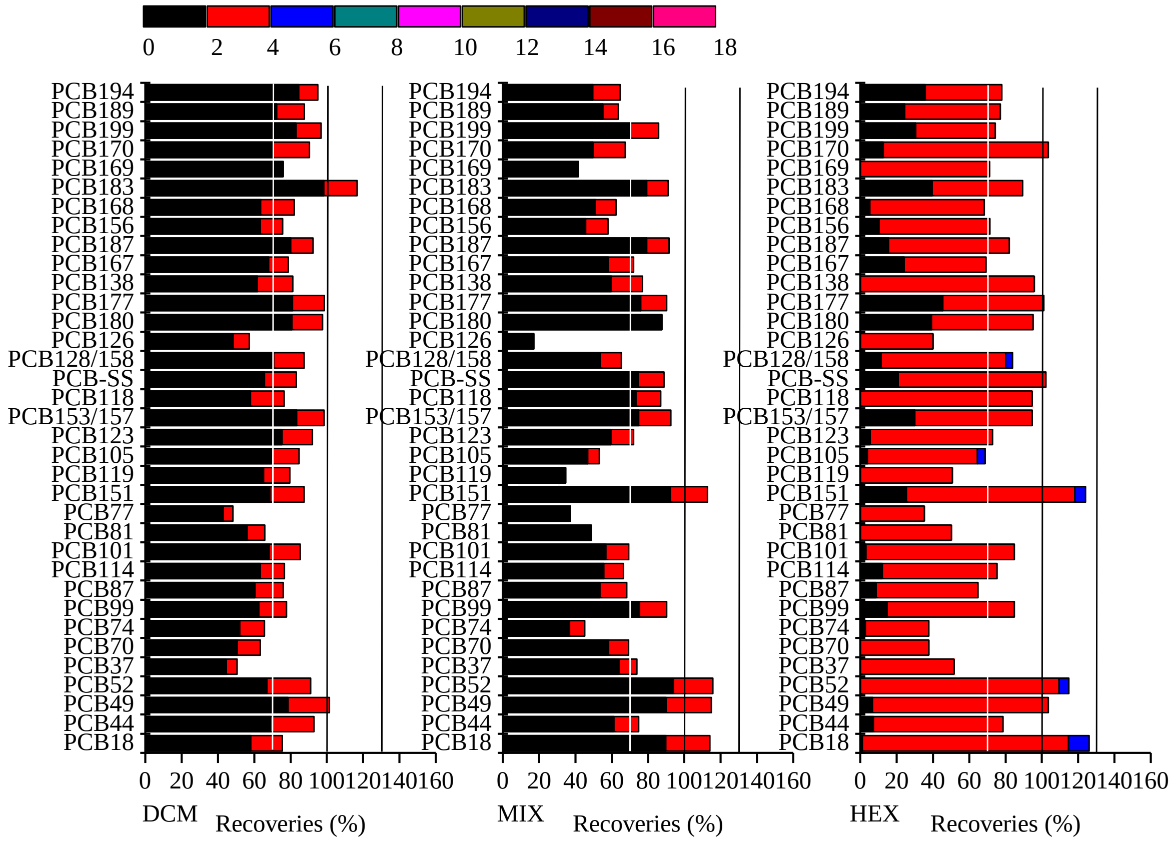

Fig. S4 Recoveries of PCBs at each SPE eluate (MIX – DCM/HEX (1:1, v/v))
